# Systems Pharmacology Approach and Experiment Evaluation Reveal Multidimensional Treatment Strategy of LiangXueJieDu Formula for Psoriasis

**DOI:** 10.3389/fphar.2021.626267

**Published:** 2021-06-08

**Authors:** Jingxia Zhao, Yan Wang, Weiwen Chen, Jing Fu, Yu Liu, Tingting Di, Cong Qi, Zhaoxia Chen, Ping Li

**Affiliations:** ^1^ Beijing Hospital of Traditional Chinese Medicine, Capital Medical University, Beijing, China; ^2^ Beijing Key Laboratory of Clinic and Basic Research with Traditional Chinese Medicine on Psoriasis, Beijing Institute of Traditional Chinese Medicine, Beijing, China; ^3^ Beijing University of Chinese Medicine, Beijing, China

**Keywords:** traditional Chinese medicine, systems pharmacology, psoriasis, liangxuejiedu formula, inflammation cytokines

## Abstract

Clinical studies have demonstrated the anti-psoriatic effect of the LiangXueJieDu (LXJD) herbal formula. However, the systemic mechanism and the targets of the LXJD formula have not yet been elucidated. In the present study, a systems pharmacology approach, metabolomics, and experimental evaluation were employed. First, by systematic absorption-distribution-metabolism-excretion (ADME) analysis, 144 active compounds with satisfactory pharmacokinetic properties were identified from 12 herbs of LXJD formula using the TCMSP database. These active compounds could be linked to 125 target proteins involved in the pathological processes underlying psoriasis. Then, the networks constituting the active compounds, targets, and diseases were constructed to decipher the pharmacological actions of this formula, indicating its curative effects in psoriasis treatment and related complications. The psoriasis-related pathway comprising several regulatory modules demonstrated the synergistic mechanisms of LXJD formula. Furthermore, the therapeutic effect of LXJD formula was validated in a psoriasis-like mouse model. Consistent with the systems pharmacology analysis, LXJD formula ameliorated IMQ-induced psoriasis-like lesions in mice, inhibited keratinocyte proliferation, improved keratinocyte differentiation, and suppressed the infiltration of CD3+ T cells. Compared to the model group, LXJD formula treatment remarkably reduced the expression of inflammatory cytokines and factors, such as IL-1β, IL-6, TNF-α, Cox2, and inhibited the phosphorylation of p-P65, p-IқB, p-ERK, p-P38, p-PI3K, p-AKT, indicating that LXJD formula exerts its therapeutic effect by inhibiting the MAPK, PI3K/AKT, and NF-қB signaling pathways. The metabolic changes in the serum of psoriasis patients were evaluated by liquid chromatography coupled with orbitrap mass spectrometry (LC-MS). The LXJD formula improved two perturbed metabolic pathways of glycerophospholipid metabolism and steroid hormone biosynthesis. Overall, this study revealed the complicated anti-psoriatic mechanism of LXJD formula and also offered a reliable strategy to elucidate the complex therapeutic mechanism of this Chinese herbal formula in psoriasis from a holistic perspective.

## Introduction

Psoriasis is an immune-mediated, chronic skin condition that follows a relapsing and remitting course. It poses a significant public health challenge with a high prevalence rate of 0.09–11.43% worldwide and multiple co-morbidities, including arthritis, cardiovascular disease, obesity, diabetes mellitus, reduced quality of life, and depression/anxiety (World health organization, 2016). Psoriasis vulgaris is the most common form of psoriasis, encompassing about 80% of psoriasis patients. Clinical manifestations comprise well-demarcation, scaling, and erythematous plaques that appear anywhere on the body. Histologically, psoriasis is characterized by parakeratosis, acanthosis with downward elongation of rete ridges, thin/no granular cell layer, Munro’s microabscesses (neutrophils in parakeratotic scale), increased mitotic figures above basal layer, and mixed dermal infiltrate of lymphocytes and neutrophils.

Current treatments for psoriasis are categorized as follows: topical therapies (topical corticosteroids, tar-based preparations, dithranol, vitamin D analogs, salicylic acid, and topical retinoids), oral medication (methotrexate, acitretin, and cyclosporine), biological agents (infliximab, adalimumab, etanercept, and ustekinumab), and Ultraviolet phototherapy (UV-B and psoralen-UV-A) (Heng et al., 2000; Gamret et al., 2018). However, 52.3% of patients with psoriasis reported dissatisfaction with their medical treatment because of the inefficacy of the methods and adverse effects (Kurd et al., 2008). Thus, the utilization of complementary and alternative medicine among psoriasis patients is common, with prevalence varying between 42 and 69% (Ben-Arye et al., 2003; Damevska et al., 2014; Talbott and Duffy, 2015). These methods constitute herbal therapies and traditional Chinese medicine (TCM) that have gained popularity in recent years. The application of TCM in the treatment of psoriasis has a long history. According to the theory of TCM, psoriasis vulgaris is classified as three main blood-related syndromes: blood heat, blood stasis, and blood dryness. The blood-heat syndrome is frequently observed at the active stage, accounting for 53.8% of the condition in psoriasis vulgaris (Zhang et al., 2009). LiangXueJieDu (LXJD) formula is commonly used to treat psoriasis vulgaris with blood-heat syndrome and has proven to be a safe remedy with better efficacy compared to conventional therapy in a multicenter randomized controlled study (Sun et al., 2020). However, the mechanisms underlying LXJD formula-alleviated psoriasis remain unclear.

TCM is based on its own theory of medicine with multiple ingredients’ interactions. Relative to Western medicine, TCM treats the function and dysfunction of the body using a holistic approach. However, the complexity of the ingredients and their *in vivo* activity could interfere with identifying the targets and understanding the mechanisms of TCM. Systems pharmacology is an emerging systematic methodology that combines oral bioavailability screening, multiple drug targets prediction and validation, and network pharmacology, providing a holistic approach to explore the targets and mechanisms of TCM. In recent years, systems pharmacology has been widely used to reveal the potential mechanism of TCM formulas (Liu et al., 2016; Yuan et al., 2020).

Based on the proved clinical efficacy, we applied the systems pharmacology method to explore the pharmacological targets and mechanisms of LXJD formula in the treatment of psoriasis in this study. Next, we verified the mechanism of LXJD formula on the main targets and those integrated by systemic pharmacology in the samples from psoriasis patients and psoriasis-like mice.

## Materials and Methods

### Reagents

High performance liquid chromatography (HPLC)-grade acetonitrile and methanol were purchased from Merck (Darmstadt, Germany). Purified water (18.2 MΩ) was obtained from a Milli-Q water purification system (Millipore, United States). Formic acid was obtained from Sigma-Aldrich (St. Louis, United States). Chlorogenic acid, caffeic acid, paeoniflorin, taxifolin, astilbin, albiflorin, resveratrol, rosmarinic acid, 5-O-methylvisammioside, quercetin, naringenin, luteolin, kaempferol, and isorhamnetin (all 98%, pure) were purchased from the National Institutes for Food and Drug Control (Beijing, China) (Table 1). All other chemicals were of analytical grade. CD3, Ki67, and loricrin antibodies were purchased from Abcam (Cambridge, MA, United States).

**TABLE 1:** Identification of the chemical constituents of LXJD formula by UHPLC-ESI-MS analysis.

No	Retention time (min)	Selected ion	Measured mass (m/z)	Error (ppm)	Identification	Formula	Chemical structure
1	20.41	(M−H)	353.0878	−0.02	Chlorogenic acid	C_16_H_18_O_9_	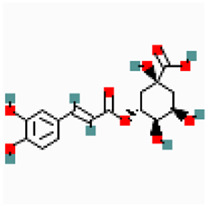
2	20.68	(M−H)	179.0351	0.63	Caffeic acid	C_9_H_8_O_4_	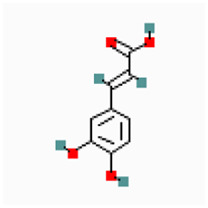
3	21.24	(M + H)	481.1712	−0.63	Paeoniflorin	C_23_H_28_O_11_	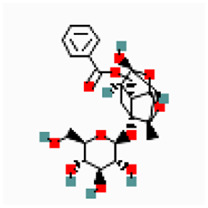
4	23.90	(M−H)	303.0510	−0.001	Taxifolin	C_15_H_12_O_7_	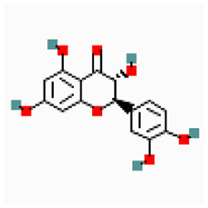
5	25.23	(M−H)	449.1090	0.03	Astilbin	C_21_H_22_O_11_	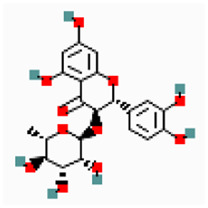
6	25.95	(M + H)	481.1713	−0.56	Albiflorin	C_23_H_28_O_11_	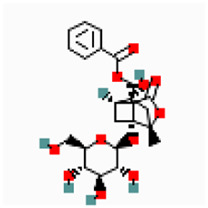
7	26.43	(M−H)	227.0714	−0.08	Resveratrol	C_14_H_12_O_3_	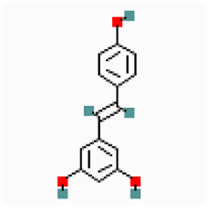
8	26.72	(M-H)	359.077	0.76	Rosmarinic acid	C_18_H_16_O_8_	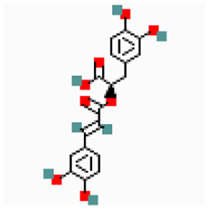
9	27.74	(M + H)	453.1760	1.04	5-O-methylvisammioside	C_22_H_28_O_10_	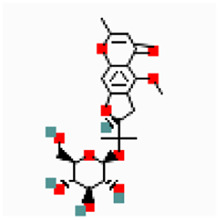
10	29.45	(M−H)	301.0352	−0.63	Quercetin	C_15_H_10_O_7_	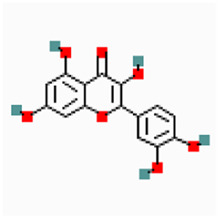
11	29.58	(M−H)	271.0612	−0.03	Naringenin	C_15_H_12_O_5_	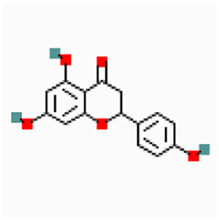
12	30.23	(M−H)	285.0403	−0.73	Luteolin	C_15_H_10_O_6_	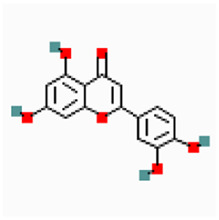
13	31.24	(M−H)	285.0404	−0.20	Kaempferol	C_15_H_10_O_6_	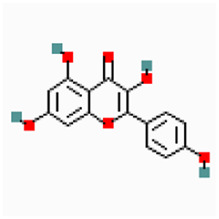
14	31.66	(M−H)	315.0510	−0.10	Isorhamnetin	C_16_H_12_O_7_	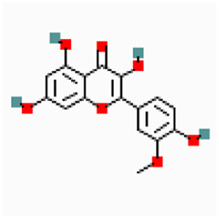

### Database Construction

A total of 864 chemical ingredients of 12 herbs in LXJD formula were collected manually from PubMed, the China National Knowledge Infrastructure (CNKI), and the Traditional Chinese Medicines Systems Pharmacology (TCMSP) databases (http://lsp.nwsuaf.edu.cn/) (Ru et al., 2014).

### Screening of Active Compounds

We employed an *in silico* integrative model-ADME (absorption, distribution, metabolism, and excretion of drugs) to screen out the compounds with favorable pharmacokinetics properties. Oral bioavailability (OB) is one of the most desirable attributes of new drugs representing the ratio of the orally administered dose that reaches the circulation system with unaltered activity.

In the present study, the OBioavail1.1 model was utilized to estimate the OB values. The compounds from LXJD formula which satisfy the criteria of OB ≥ 30% were selected as candidate active molecules for further screening (Ru et al., 2014; Yuan et al., 2020). Drug-likeness (DL) is a major factor in the absorption, distribution, metabolism, and elimination of the molecules in the human body. It is estimated from the molecular structure before the substance is synthesized and tested. Herein, a compound with DL ≥ 0.18 was selected as the active compound of herbs for subsequent studies (Ru et al., 2014; Yuan et al., 2020). The oral absorption of drugs was realized *via* intestinal epithelial cells (IECs). In this study, a computer Caco-2 permeability prediction model was applied to predict the intestinal permeability of the components in TCMSP (Lv et al., 2019). The chemical ingredients with Caco-2 cell permeability ≥–0.4 were filtered out as candidate active compounds. The oil-water partition coefficient AlogP is the logarithm of the ratio of the partition coefficient of the substance in n-octanol and water. Lipophilicity is a leading physical and chemical parameter reflecting the permeability in biofilms. ALOGPS2.1 software was used to calculate AlogP, and the threshold was set to <5 according to Lipinski’s rule of five (Lipinski et al., 2001; Tetko and Bruneau, 2004).

### Target Fishing

A weighted ensemble similarity (WES) model was implemented to predict the potential target molecules of the candidate active compounds in LXJD formula. Then, a similarity search based on chemical fingerprinting was applied to obtain potential targets (http://sea.bkslab.org/search/). Finally, the targets from different sources were analyzed against the Pharmacogenomics knowledgebase (PharmGKB, https://www.pharmgkb.org/), Therapeutic Targets Database (TTD, http://database.idrb.cqu.edu.cn/TTD/), and Comparative Toxicogenomics Database (CTD, http://ctdbase.org/) to eliminate redundant and erroneous molecules, thereby guaranteeing the accuracy of the database.

### Gene Ontology Enrichment and Clustering Analysis for Targets

To identify the targets associated with the physiological features of psoriasis, a Gene Ontology (GO) enrichment analysis was performed using DAVID (http://david.abcc.ncifcrf.gov) for classification. In this section, the GOBP (biological process) analysis result was highlighted. Then, functional annotation clustering analysis was carried out based on the GOBP enrichment data. *p*-value <0.05 indicated statistical significance.

### Network Construction

TCM is a complex system consisting of multiple effective compounds, multiple targets of action, and related diseases. To illustrate the pharmacological mechanisms of LXJD formula in psoriasis treatment and clarify the complicated associations among the active compounds, targets, and diseases at a systems level, the network visualization analysis Cytoscape software was used to establish the compound-target network, target-disease network, and target-pathway network, respectively. In order to further interpret the molecular mechanism of LXJD formula against psoriasis, an integrated map of “psoriasis-related pathways” was constructed by integrating the key pathway obtained from the Kyoto Encyclopedia of Genes and Genomes (KEGG) database (http://www.kegg.jp/) using target-pathway network analysis.

### Preparation of LiangXueJieDu Formula

LXJD formula, consisting of Tu Fu Ling (*Smilax glabra* Roxb.) (ratio 1:5), Huai Hua (Styphnolobium japonicum (L.) Schott syn. Sophora japonica L.) (ratio 1:10), Di Huang (Rehmannia glutinosa (Gaertn.) DC.) (ratio 1:10), Zi Cao (Arnebia euchroma (Royle ex Benth.) I.M.Johnst.) (ratio 1:15), Quan Shen (Bistorta officinalis Delarbre syn. Polygonum (Isatis tinctoria L.) (ratio 1:15), Bai Mao Gen (Imperata cylindrica (L.) P.Beauv.) (ratio 1:15), Bai Xian Pi (Dictamnus dasycarpus Turcz.) (ratio 1:15), Bai Hua She She Cao (Scleromitrion diffusum (Willd.) R.J.Wang syn. Hedyotis diffusa Willd.) (ratio 1:15), Fang Feng (Saposhnikovia varzcata (Turcz.) Schischk.) (ratio 1:15), and Mu Dan Pi (Paeonia x suffruticosa Andr), were decocted and provided by the TCM Pharmacy of Beijing Hospital of Traditional Chinese Medicine. Briefly, 150 g of the crude drugs of LXJD formula were soaked and decocted in 400 ml of pure water for 30 min. Then, the water decoction was concentrated to 200 ml, and the final dosage of crude drugs was 0.75 g/ml.

All raw herbs were purchased from Beijing Xinglin Pharmaceutical (Beijing, China, Voucher number 20150301). The voucher specimen, identified by Professor Wei He, was deposited in our laboratory at Beijing Traditional Chinese Medical Hospital.

### Preparation of Standard Solutions and Liquid Chromatography

A standard working solution of the 14 reference substances (Table 1, purities>98%) were prepared in concentrations ranging from 0.032 to 0.058 mg/ml in methanol and stored at 4°C. The 14 reference substances were used as standard control to identify the major components of LXJD formula by the UPLC-MS method.

The UPLC separation was performed on an ACQUITY UPLC^™^ BEH C18 column (100 × 2.1, 1.7 mm). Both negative and positive modes were examined. A gradient elution was used with 0.1% formic acid in methanol as solvent A and 0.1% formic acid in water as solvent B, at a flow rate of 0.2 ml min^−1^. The following gradient elution scheme was used: 0–2 min, 100–100% B; 2–10 min, 100–93% B; 10–40 min, 93–0% B; 40–45 min, 0–0% B; 45–45.1 min, 0–100% B; and 45.1–60 min, 100–100% B. The injection volume was 10 μl.

### Mass Spectrometric Conditions

A Thermo Q Exactive^™^ Plus Orbitrap^™^ mass spectrometer was used for the qualitative analysis of the constituents in LXJD formula. Mass spectrometric parameters were: sheath gas flow rate of 35; aux gas flow rate of 10; sweep gas flow rate of 0; S-lens RF level of 50. The spray voltage was 3.5 kV for the positive and −4.5 kV for the negative ion mode. The capillary temperature and aux gas heater temperature were set to 320 and 350 °C, respectively. The m/z ranged from 100–1200 and 150–2000 for the positive and negative ion modes, respectively.

### Study Design

A total of 40 psoriasis patients were recruited from the Beijing Traditional Chinese Medical Hospital according to the specified inclusion and exclusion criteria.

The inclusion criteria were as follows: 1) Psoriasis vulgaris was diagnosed according to the “Clinical Guidelines of Psoriasis 2008” reported by the Chinese Medical Association; 2) age 18–65 years; 3) willing to sign a written informed consent; 4) psoriasis patients were diagnosed with blood-heat syndrome based on the theory of TCM; 4) PASI score >5.

Exclusion criteria (patients who met one of the following conditions were excluded): 1) Those who were allergic to LXJD formula or the composition; 2) women who were pregnant or in the lactation stage; 3) those who had been treated with steroids orally in the past 2 weeks or with retinoid orally or steroid topically in the past week; 4) arthropathic, pustular, or erythrodermic psoriasis; 5) those with severe heart, cerebrovascular, liver, kidney, hematopoietic system, cancer, or psychosis diseases.

The treatment group received the Chinese herbal medicine LXJD formula for 8 weeks. Each dose of LXJD formula (150 g of crude drugs) was decocted in 400 ml of water. Then, the water decoction was concentrated to 200 ml, and the final dosage of crude drugs was 0.75 g/ml. In total, 100 ml of the decoction was taken orally 30 min before breakfast and the other 100 ml 30 min before dinner. Serum samples were collected from each patient before and 8 weeks after LXJD formula treatment.

The study duration was 8 weeks. Patients were evaluated before the start of treatment and were followed up every 2 weeks during the trial period. Clinical symptoms, examination of psoriatic skin lesions, and psoriasis area and severity index (PASI) scores were documented. The PASI is a composite index of the severity of the three main characteristics of psoriatic plaques (erythema, scaling, and thickness) weighted by the amount of coverage of these plaques in the four main body areas (i.e., head, trunk, upper extremities, and lower extremities). PASI scores can range from 0 to 72, wherein higher scores indicate greater severity.

Out of a total of 40 potentially eligible patients, 15 patients were subsequently excluded from this study, of which 7 patients were excluded from the Chinese medicine treatment group for failing to adhere to the clinical trial protocol and eight patients were excluded because of the progression and evolution of patients’ symptoms.

Of the remaining 25 patients, 52% of patients (13 in 25) after LXJD treatment achieved PASI60 and were considered as responders to LXJD treatment. Serum metabolomics were analyzed in these 13 responders’ samples only.

### Ethics Statement

Informed consent was given by the patients and this study was approved by the Ethics Committee of Beijing Traditional Chinese Medical Hospital (NO. 2017BL-073–01).

### Sample Preparation

Serum (100 μl) was added into methanol (400 μl), vortex-mixed for 30 s, and the proteins were precipitated by centrifugation at 12,000 rpm for 15 min. Subsequently, the dried residue was reconstituted in 100 μl of acetonitrile-water (10:90, v/v) and then injected for LC-MS analysis.

### Metabolic Profiling

The chromatographic analysis of the serum sample was performed in a Thermo LC system (Thermo, Ultimate 3000 LC, Orbitrap Elite) equipped with a Hypergod C18 column (4.6 mm × 100 mm, 3.0 μm). The column was maintained at 40°C, and the flow rate was set at 0.30 ml/min. The mobile phase was composed of water (A) and acetonitrile (B), each containing 0.1% formic acid. The linear gradient program was optimized as follows: 0–2.0 min, 5% B; 2.0–12 min, 5–95% B; 12–15 min, washing with 95% B for 15–17 min, and equilibration with 5% B. A volume of 4 μl was injected in all the cases and analyzed on a Thermo mass spectrometer equipped with an electrospray ionization source (ESI) operating in positive and negative ion modes. The optimized parameters of TOF were set as follows: the spray voltage was 3.0 kV (ES+) and 3.2 kV (ES−) with the capillary temperature at 350°C, and sheath gas flow and auxiliary gas flow were set at 45 and 15, respectively. The mass range was set at 100–1000 m/z with 70,000 mass resolution at 400 m/z. The accepted scientific names must be updated using Medicinal Plant Name Service - KEW (https://mpns.science.kew.org/mpns-portal/).

The MS raw data of serum samples were processed using SIEVE software (Thermo Corp., Manchester, United Kingdom). This process included deconvolution, normalization, and alignment, and a dataset including retention time and mass pairs with the corresponding intensities of each metabolite was obtained. Then, the processed dataset was introduced into the SIMCA-P software package (v13.0, Umetric, Umeå, Sweden) to perform principal component analysis (PCA) and orthogonal to partial least squares-discriminate analysis (OPLS-DA). In the OPLS-DA model, the variable importance of project (VIP) value and *S*-plot were employed as standards to select potential biomarkers.

### Animals

Eight-week-old male C57BL/6 mice were purchased from Beijing Huafukang Biological Technology Co., Ltd. (Beijing, China) and maintained under specific pathogen-free conditions. All experiments adhered to the principles of the Declaration of Helsinki and were conducted using the protocols approved by the Beijing Institute of Traditional Chinese Medicine.

### Establishment of Imiquimod (IMQ)-Induced Psoriasis-like Mouse Models

Mice were divided into four groups (10 mice/group): Control group, model group, MTX group, and LXJD group. All mice except those in the control group received a daily topical application of 42 mg of IMQ cream (5%) (MedShine, Chengdu, China) on their shaved back for seven consecutive days to establish a model of psoriasiform lesions. LXJD and MTX treatment started with the application of IMQ. From days 1–7, mice in the LXJD and MTX (positive control) groups were treated with LXJD (29.4 g herb/kg/day) and MTX (1 mg/kg/day) by gavage, respectively. Mice in the control group (control) and model group (model) were given saline by intragastric administration. On day seven, the animals were sacrificed, and skin samples were collected from their backs.

### Histology, Immunohistochemistry, and Immunofluorescence Staining

The mouse back skin was fixed in formalin and embedded in paraffin. Partial sections were stained with hematoxylin-eosin (H&E) for pathological observation by microscopy. Partial sections were stained with anti-Rabbit CD3 (1:100, Abcam, United States) and anti-Loricrin (1:200, Abcam, United States), and Diaminobezidin (DAB) (Zhongshan Golden Bridge Biotechnology, China) was used for color development. For immunofluorescence, Ki-67 (proliferation) level was evaluated in skin sections using anti-Ki67 antibody (1:200, Abcam), following the manufacturer’s instructions. For CD3^+^, Ki-67+, and Loricrin+ cells, three areas in three sections of each sample were selected randomly, and the number of positive cells was calculated.

### Quantitative RT-PCR Analysis (qRT-PCR)

Total RNA was extracted from the skin lesion and cultured cells using an ultrapure RNA extraction kit (Kang Biotechnology, China). Reverse transcription was carried out using the HiFi-MMLV cDNA first-strand synthesis kit (Kang Biotechnology). Quantitative real-time PCR amplification was performed on ABI 7500 Real-Time PCR Systems (Applied Biosystems, United States). A housekeeping gene was utilized as an internal reference standard. The relative gene expression was calculated using the 2-^ΔΔCt^ method.

### Western Blot

The tissue extracts were prepared using RIPA buffer (50 mM of Tris-HCl pH 7.4, 150 mM of NaCl, 1 mM of EDTA, 1% Triton X-100, 0.1% SDS, 0.5% deoxycholate) supplemented with a complete protease inhibitor cocktail (Roche) and a PhosSTOP phosphatase inhibit or cocktail (Roche). Samples were subjected to SDS-PAGE and the resolved proteins were then transferred onto a polyvinylidene difluoride (PVDF) membrane (Millipore). The immunoblots were probed with indicated antibodies and visualized using a SuperSignal West Pico chemiluminescence ECL kit (Pierce). GAPDH was used as an internal control.

### Statistical Analysis

All quantitative data are presented as means ± SD and analyzed for statistical significance using GraphPad Prism 5.0 (GraphPad Software Inc., San Diego, CA, United States). One-way analysis of variance (ANOVA) followed by Tukey’s post hoc test and unpaired Student’s t-test was used to analyze the statistical significance among multiple groups and between two groups, respectively. A two-tailed Student’s *t*-test was performed in a clinical study using the Statistical Package for Social Science program (SPSS 20.0, Chicago, IL, United States). *p* < 0.05 indicated statistically significant difference.

## Results

### Bioactive Compounds Identification for Each Herb in LiangXueJieDu Formula

In the current study, 864 chemical ingredients and their pharmaceutical properties in the LXJD formula were obtained from the TCMSP database. A combination of OB (≥30%), Caco-2 permeability (Caco-2) (>−0.4), prediction of permeability (AlogP <5), and drug-likeness (DL) ≥0.18 properties was applied to identify the active compounds of LXJD formula. Based on a previous report, some compounds that did not agree with the threshold of compounds screening were also likely to produce therapeutic effects, and hence, retained as active compounds in this study, such as palmitic acid (Dogra et al., 2018), sitosterol (REISS and JAIMOVICH, 1958), stigmasterol (Srivastava, 2014), trametenolic acid (Ma et al., 2013), lignoceric acid (Tsoukalas et al., 2019), tirucallane (Mireku et al., 2015), etc. These compounds have bioactivity and are reported as anti-psoriatic or anti-inflammatory molecules. Finally, 144 active compounds (Supplementary Table S1), including 37 in Isatis tinctoria, 8 in Scleromitrion diffusum, 10 in Dictamnus dasycarpus, 9 in Paeonia lactiflora, 11 in Rehmannia glutinosa, 14 in Arnebia euchroma, 3 in Paeonia x suffruticosa, 18 in Saposhnikovia varzcata, 5 in Smilax glabra, 8 in Imperata cylindrica, 7 in Bistorta officinalis, and 14 in Paeonia lactiflora, were obtained*.*

### Target Identification and Analysis

TCM formulas exert their pharmacological activities through synergistic interactions between multiple compounds and targets. To identify the relevant targets of the potential compounds in LXJD formula, the TCMSP database and WES algorithms were used. A final list of 125 potential targets for 144 bioactive compounds was obtained. These targets were further subject to PharmGkb, CTD, and TTD for sanity checks. As shown in Supplementary Table S2, most active compounds could act on multiple targets simultaneously, and one target could also be linked to multiple active compounds. PTGS2, also known as cyclooxygenase-2 (COX-2), is one of the potential targets that play a critical role in inflammation (Yalçin et al., 2007). It was predicted that PTGS2 is linked to 86 active compounds in the LXJD formula to exert therapeutic effect synergistically. The active compound quercetin (MOL60) was found to be connected to 79 targets, reflecting the multiple targets of herbal medicine.

### Gene Ontology Enrichment Analysis for Potential Targets

**FIGURE 1:**
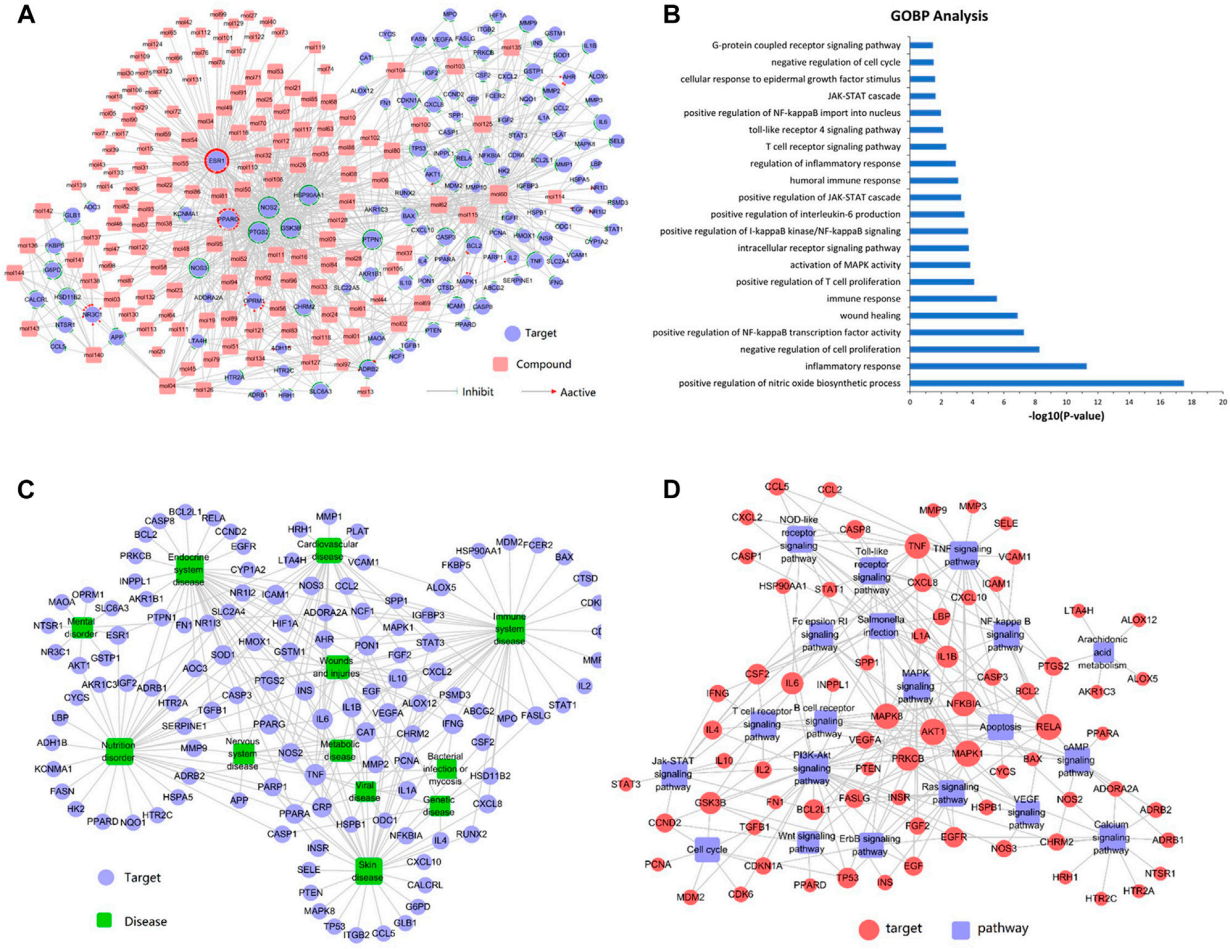
**(A)** Compound-target network analysis. A compound and a target are linked if the target protein is hit by the corresponding compound. Node size is proportional to its degree. The pink square represents the compounds and the purple circle represents the targets. **(B)** GO enrichment analysis of the predicted protein targets. The top 21 enriched biological processes related to the targets are shown (*p* < 0.05). **(C)** Target-disease network. Target proteins are linked to their corresponding diseases and those diseases are linked to the corresponding disease categories they belong to. The purple circle represents the target and the green square represents the disease. **(D)** The target-pathway network was constructed by linking active compounds to their related pathways. Node size is related to the degree. The red circle represents the target and the purple square represents the pathway.

To validate whether the 125 potential targets in the network are associated with psoriasis, we performed GO term (BP) enrichment analysis. The top 21 significant GO terms responsible for psoriasis were enriched. As shown in Figure 1B, inflammatory response, positive regulation of NF-қB transcription factor activity, positive regulation of T cell proliferation, regulation of inflammatory response, and IL-6 production was intricately linked to the pathogenesis of psoriasis. These results suggested that the potential targets were significantly involved in the pathogenesis of psoriasis.

### Compound-Target Network Analysis

Figure 1A shows that the compound-target network consisted of 144 active compounds, 125 targets, and 901 compound-target interactions (269 nodes and 901 edges). The results displayed an average of 7.2° per compound and 6.3° per target protein, respectively. In the correlations between compounds and targets (Supplementary Table S3), MOL60 exhibited the highest number of target interactions (79°), followed by MOL125 (35°), MOL115 (34°), and MOL62 (33°), which was consistent with the multi-component and multi-target traits of herbal medicines. Regarding the candidate targets, estrogen receptor (ESR1) showed the highest degree (113°), followed by PTGS2 (86°), NOS2 (81°), PPARG (60°), and GSK3B (56°), which demonstrated the potential therapeutic effect of each drug in LXJD formula for ameliorating psoriasis.

### Target-Disease Network

In order to elucidate the correlation between LXJD formula and diseases, a target-disease network, including 122 target proteins and 12 corresponding diseases, was constructed based on the DrugBank, TTD, and PharmGKB databases. As shown in Figure 1C, LXJD formula was related to 12 types of diseases, including immune system disease (41°), endocrine system disease (37°), skin disease (35°), nutrition disorder (35°), cardiovascular disease (27°) wounds and injuries (29°), metabolic disease (14°), nervous system disease (10°), mental disorder (9°), genetic disease (7°), viral disease (7°), and bacterial infection or mycosis (4°). The target-disease network might explain the theory of “homotherapy for heteropathy” in TCM.

### Target-Pathway Network Analysis

To further elucidate the therapeutic mechanisms underlying the LXJD formula in the treatment of psoriasis, all the predicted target proteins were mapped to enrich the relevant pathways. The target-pathway network containing 73 targets and 20 corresponding pathways is shown in Figure 1D. The majority of the target proteins were involved in multiple pathways, indicating that the target proteins of the LXJD formula interplayed with each other in different pathways and exerted synergistic effects in the treatment of psoriasis. As shown in Supplementary Table S4, the crucial target protein-associated pathways included the PI3K/AKT signaling pathway (27°), TNF signaling pathway (21°), MAPK signaling pathway (16°), toll-like receptor signaling pathway (15°), NOD-like receptor signaling pathway (13°), *salmonella* infection pathway (13°), Ras signaling pathway (13°), apoptosis pathway (12°), calcium signaling pathway (12°), NF-қB signaling pathway (11°), T cell receptor signaling pathway (11°), and JAK-STAT signaling pathway (11°). These results suggested that multiple targets affect various pathways regulating the pathological processes underlying psoriasis, thereby promoting a potent therapeutic approach for these diseases.

### Psoriasis-Related Pathway Analysis

**FIGURE 2:**
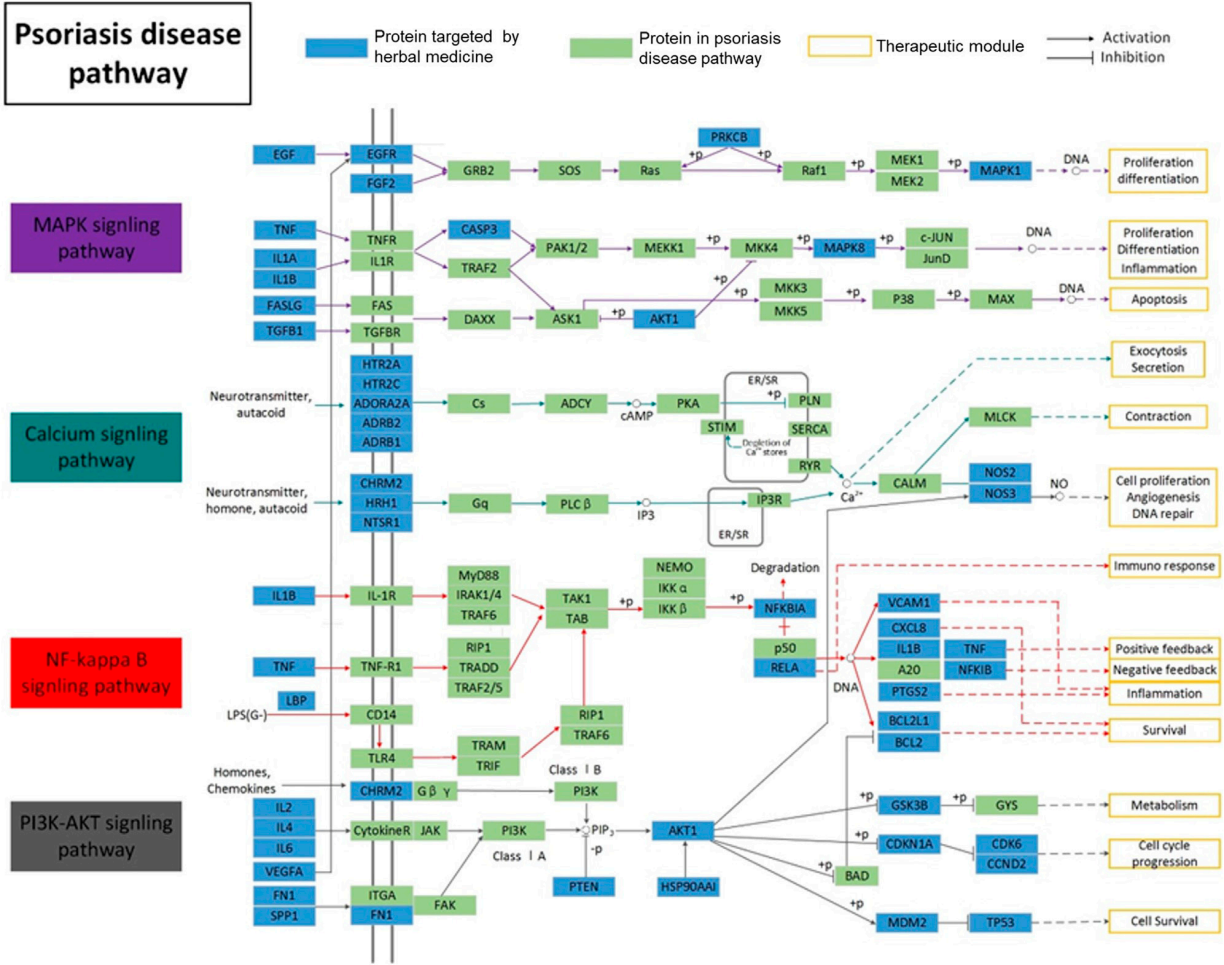
Distribution of target proteins of LXJD formula in integrated psoriasis-related pathways, which includes the MAPK, calcium, NF-κB, and PI3K/AKT signaling pathways.s

Based on the complex mechanism of the LXJD formula in the treatment of psoriasis, an integrated map of “psoriasis-related pathways” was constructed by integrating the key pathways obtained from the KEGG database using target-pathway network analysis. As shown in Figure 2, psoriasis-related pathways consist of four important pathways: the MAPK signaling pathway, calcium signaling pathway, PI3K/AKT signaling pathway, and NF-қB signaling pathway. These pathways were associated with several biological functions, such as inflammation, proliferation, metabolism, cell survival, cell cycle, and immune response, indicating the therapeutic effect of the LXJD formula in psoriasis.

### Identification of Major Components of LiangXueJieDu Formula

**FIGURE 3:**
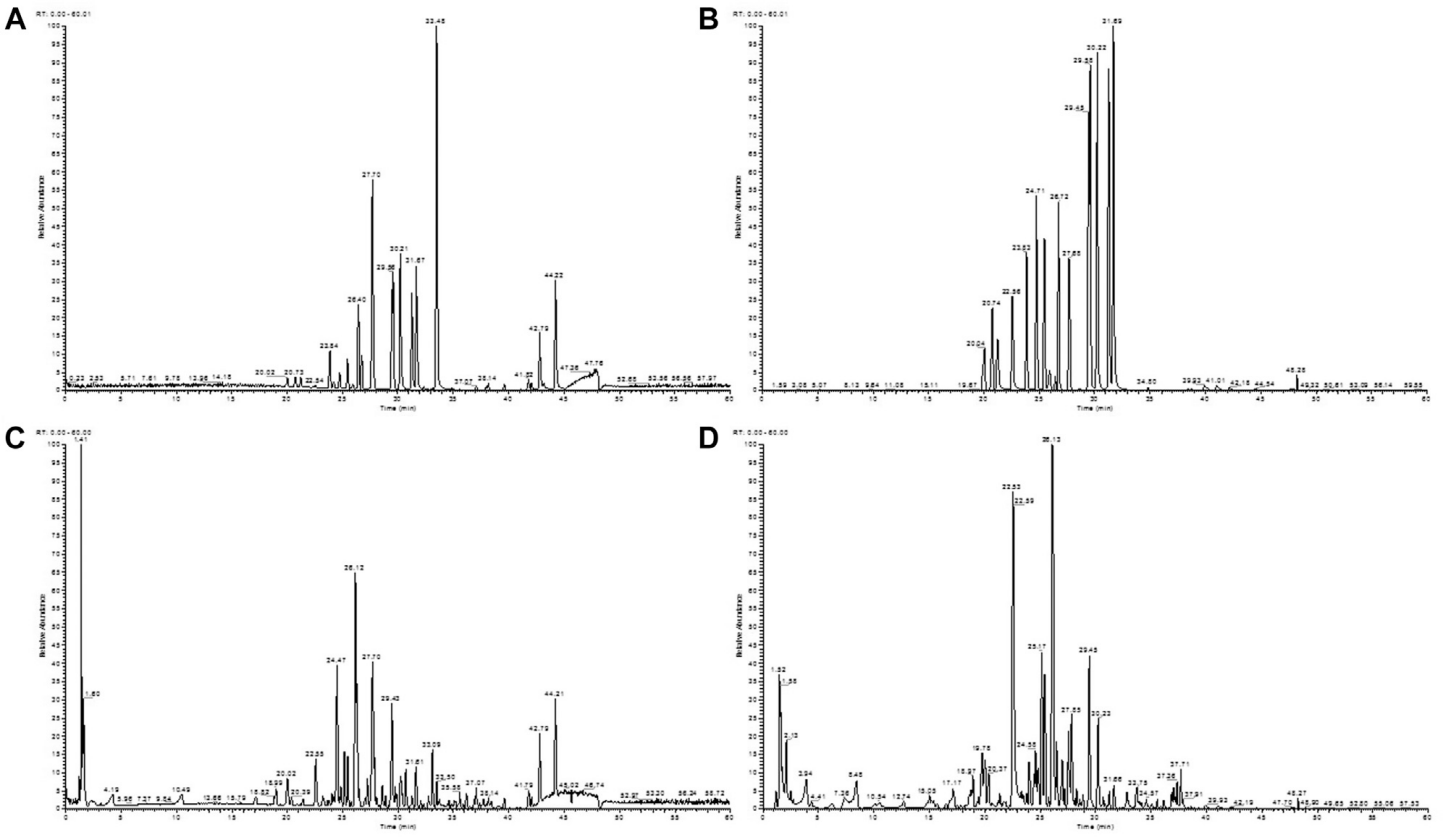
Total ion chromatograms of a mixture of 14 standards obtained from the negative **(A)** and positive **(B)** ion scan modes. LXJD formula extract obtained from the negative **(C)** and positive **(D)** ion scan modes.

The total ion chromatograms of the LXJD formula extracted in both the positive and negative ion modes are shown in Figure 3. The MS data exhibited high precision with the mass accuracy within 5 ppm. Based on the MS spectra and fragmentation pattern using public databases (Chemspider and MassBank) or literature, 14 compounds were identified (Table 1). Chlorogenic acid, caffeic acid, paeoniflorin, taxifolin, astilbin, albiflorin, resveratrol, rosmarinic acid, 5-O-methylvisammioside, quercetin, naringenin, luteolin, kaempferol, and isorhamnetin were the main components of the LXJD formula extract, which was consistent with the systems pharmacology prediction. These results also demonstrated that the UPLC-MS method was suitable for identifying these compounds.

### Effect of LiangXueJieDu Formula in Psoriasis Patients

**FIGURE 4:**
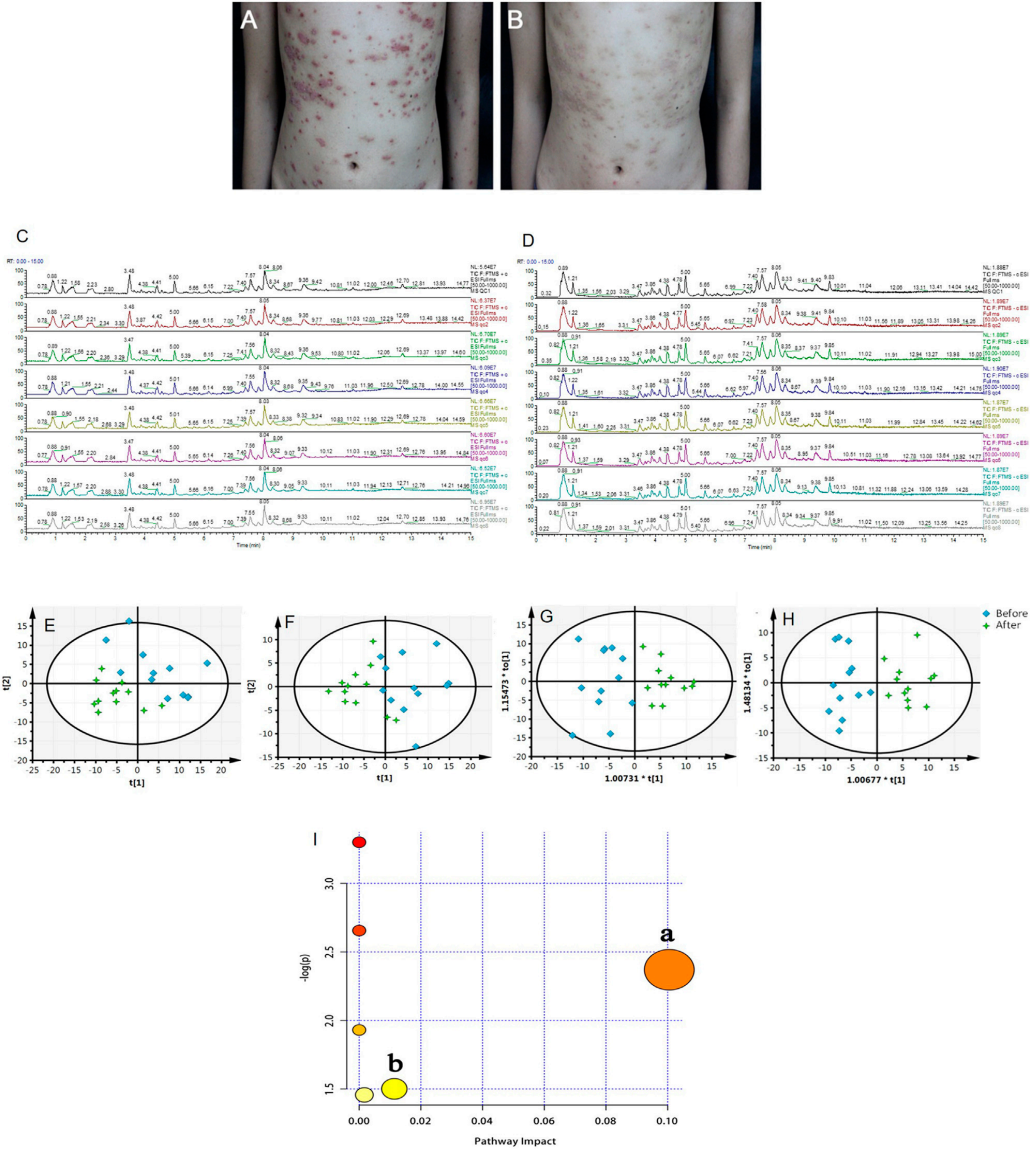
**(A, B)** Representative photographs of the patient with psoriasis taken at baseline (week 0, A) and after 8 weeks **(B)** of treatment with LiangxueJiedu formula. Total ions chromatogram of QC samples from the positive **(C)** and negative **(D)** ion scan modes. **(E, F)** PCA score plots of serum samples collected from psoriasis patients before and after 8 weeks of LiangxueJiedu formula treatment measured in **(E**, *R* ^2^X = 0.724, Q^2^ = 0.495**)** positive and **(F**, *R* ^2^X = 0.574, Q^2^ = 0.371**)** negative mode. **(G, H)** OPLS-DA score plots of serum samples collected from psoriasis patients before and after 8 weeks of LiangxueJiedu formula treatment based on UPLC-Q-TOF/MS technology in (G, *R* ^2^X = 0.557, *R* ^2^Y = 0.782, Q^2^ = 0.671) positive and (H, *R* ^2^X = 0.410, *R* ^2^Y = 0.852, Q^2^ = 0.453) negative mode. **(I)** Summary of pathway analysis with MetPA, a: glycerophospholipid metabolism; b: steroid hormone biosynthesis.

After 8 weeks of treatment with LXJD formula, the skin symptoms of all patients improved. In addition, psoriasis area and severity index (PASI) scoring, a critical index to evaluate psoriasis in the clinic, decreased significantly (*p* < 0.05) following LXJD formula treatment (Figures 4A,B and Table 2).

**TABLE 2:** Characteristics of the study cohorts.

	Before treatment	After treatment
Gender	7/6	7/6
PASI	13.6 ± 5.97	3.8 ± 2.1**

Values are reported as the mean ± SD. Units for cholesterol and triglycerides are mmol/L, PASI, psoriasis area, and severity index. Gender balance: male/female. *p* < 0.05 indicated statistical significance for PASI scores before and after treatment (**p < 0.01).

### Serum Metabolomics Study of Psoriasis Patients

Serum metabolic profiling of psoriasis patients was acquired to explore the protective effect of the LXJD formula. UPLC/Q-TOF-MS was applied for analysis. The quality-control (QC) sample was used to monitor the precision, repeatability, and reliability of the LC-MS measurements and peak intensity data (Figures 4C,D). These results indicated that methods were suitable for subsequent sample analysis. Principal component analysis (PCA) was employed to obtain unsupervized multivariate statistical analysis of serum metabolomics. In Figures 4E,F, serum metabolites in positive and negative ion modes were displayed by score plots, the cluster of psoriasis patients before LXJD formula treatment was clearly separated from those after 8 weeks of LXJD formula treatment in both modes. The OPLS-DA models were established to identify the discriminating ions contributing to the classification before and after LXJD formula treatment patients (Figures 4G,H and Table 3). The score plots based on the serum metabolomics of these two groups in positive and negative ion modes are shown in Figures 4G,H, the values of R^2^X, R^2^Y, and Q^2^ were 0.557, 0.782, and 0.671 in the positive ion mode, 0.410, 0.852, and 0.453 in the negative ion mode, indicating the classification was well suited for the models. A total of 11 metabolites in the positive mode and seven metabolites in the negative mode were altered after LXJD formula treatment. These included cortisone (S1), L-urobilinogen (S2), PA[15:1(9Z)/22:4(7Z,10Z,13Z,16Z)] (S3), PC[18:0/18:4(6Z,9Z,12Z,15Z)] (S4), PC[18:1(9Z)/4:0] (S5), PC[20:0/14:1(9Z)] (S6), PC[20:4(8Z,11Z,14Z,17Z)/18:3(6Z,9Z,12Z)] (S7), PG[12:0/20:5(5Z,8Z,11Z,14Z,17Z)] (S8), PG[P-18:0/20:4(5Z,8Z,11Z,14Z)] (S9), phenyllactic acid (S10), traumatic acid (S11), 16-hydroxy hexadecanoic acid (S12), 4-hydroxybutanoic acid (S13), L-gamma-glutamyl-L-isoleucine (S14), MG[0:0/16:1(9Z)/0:0] (S15), PA[20:4(5Z,8Z,11Z,14Z)/18:4(6Z,9Z,12Z,15Z)] (S16), PG[20:3(8Z,11Z,14Z)/20:0] (S17), and PS[21:0/18:3(6Z,9Z,12Z)] (S18). To probe the associations between the key metabolic pathways and the regulatory effects of LXJD formula, metabolic pathway analysis (MetPA) (software of MetaboAnalyst 3.0; http://www.metaboanalyst.ca/) was performed by the integration of all serum potential metabolites. As a result, two disturbed metabolic pathways, glycerophospholipid metabolism and steroid hormone biosynthesis were considered as the most relevant pathways involved in psoriasis (Figure 4I). LXJD formula treatment improved both perturbed metabolic pathways.

**TABLE 3:** Potential metabolites characterized in the serum profile and the altered trends in different groups.

NO.	Metabolites	VIP	m/z	Rt	*p*-value	Fold-change (B/A)
S1	Cortisone	1.025	360.1936	4.487	0.018	0.268
S2	L-Urobilinogen	1.111	596.3528	7.711	0.009	−0.854
S3	PA[15:1(9Z)/22:4(7Z,10Z,13Z,16Z)]	1.187	708.4561	3.832	0.005	−0.229
S4	PC[18:0/18:4(6Z,9Z,12Z,15Z)]	1.122	781.5589	10.116	0.008	0.899
S5	PC[18:1(9Z)/4:0]	1.075	591.3974	7.707	0.012	−0.672
S6	PC[20:0/14:1(9Z)]	1.070	759.5765	11.030	0.013	0.486
S7	PC(20:4[8Z,11Z,14Z,17Z)/18:3(6Z,9Z,12Z)]	1.103	803.5414	10.122	0.010	1.014
S8	PG[12:0/20:5(5Z,8Z,11Z,14Z,17Z)]	1.031	712.4401	8.479	0.017	−0.701
S9	PG[P-18:0/20:4(5Z,8Z,11Z,14Z)]	1.159	782.5629	10.828	0.006	0.650
S10	Phenyllactic acid	1.037	166.0626	4.306	0.016	0.633
S11	Traumatic acid	1.145	228.1364	6.101	0.007	−0.489
S12	16-hydroxy hexadecanoic acid	1.015	272.2349	7.480	0.019	−0.424
S13	4-hydroxybutanoic acid	1.106	104.0476	1.624	0.010	−0.694
S14	L-gamma-glutamyl-L-isoleucine	1.004	258.1369	5.235	0.021	−0.758
S15	MG[0:0/16:1(9Z)/0:0]	1.133	328.2605	11.324	0.008	−0.576
S16	PA[20:4(5Z,8Z,11Z,14Z)/18:4(6Z,9Z,12Z,15Z)]	1.043	716.4334	8.207	0.016	−0.756
S17	PG[20:3(8Z,11Z,14Z)/20:0]	1.030	828.5686	11.208	0.017	0.481
S18	PS[21:0/18:3(6Z,9Z,12Z)]	1.048	827.5646	11.112	0.015	0.508

Potential metabolites are characterized in the serum profile and their altered trends in different groups. Fold-change (B/A) represents the changing trend before and after LXJD formula treatment based on log 2 (B/A).

### LiangXueJieDu Formula Improved the Differentiation of Keratinocytes and Alleviated the Proliferation of Keratinocytes in an Establishment of Imiquimod-Induced Psoriasis-Like Mouse Model

**FIGURE 5:**
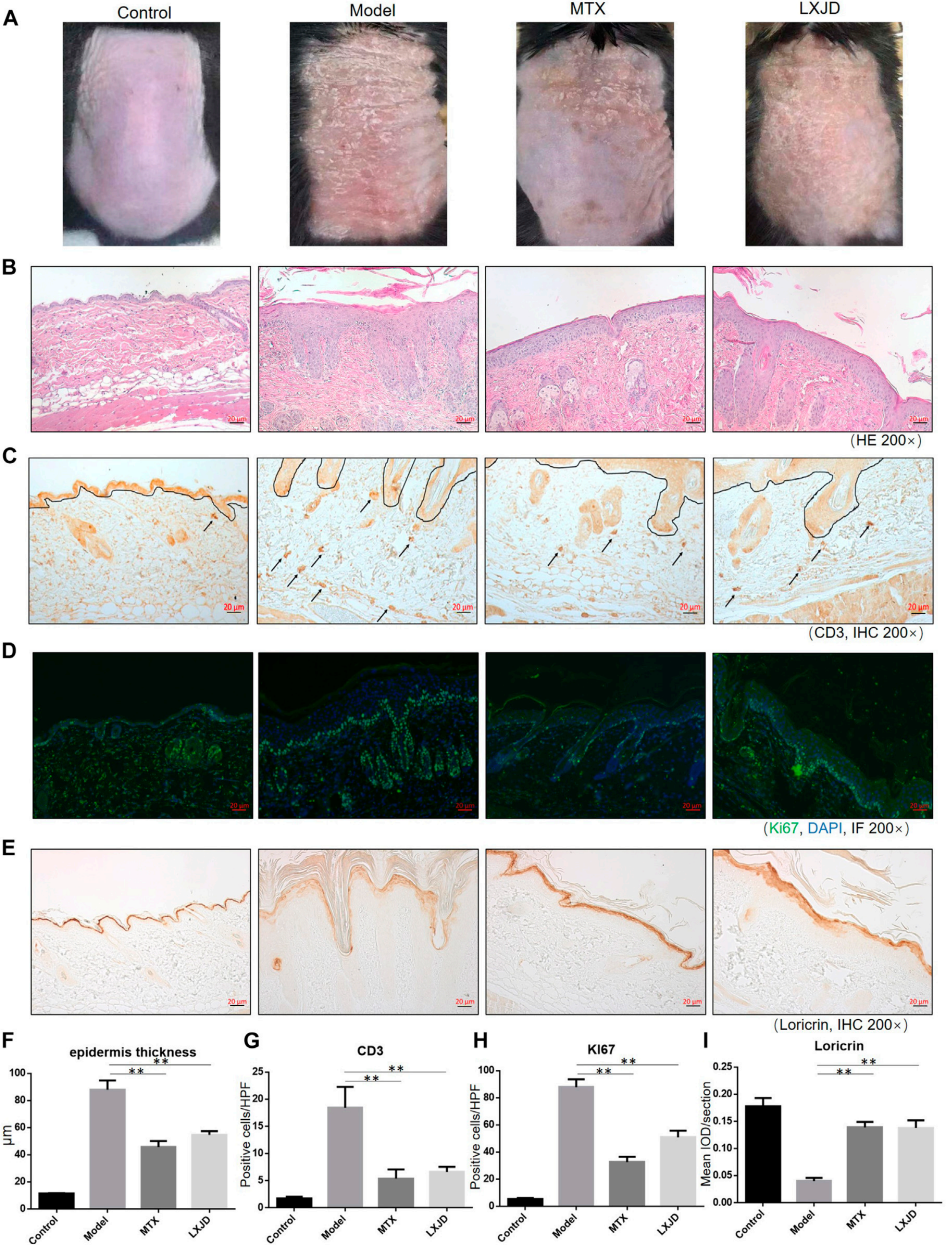
C57 BL/6 mice were treated daily with IMQ cream on the shaved back skin to induced psoriasis-like dermatitis, while the control group (CTRL) was treated with matrix. The IMQ-treated mice were administered saline (model) [methotrexate (MTX) serves as a positive control] or LXJD formula for a total of 7 days. **(A)** Phenotypic presentation of the back skin on day 7 after IMQ treatment. **(B)** Histological analyses of mouse back skin by H&E staining. **(C)** Immunohistochemical staining of CD3+ cells in skin lesions. Mouse back skin was stained for Ki67 **(D)** and loricrin **(E)**. **(F–I)** Quantification analysis for epidermis thickness, CD3+ and Ki-67+, and loricrin was performed. Data are shown as mean ± SD. n = 5 mice. *p < 0.05 and **p < 0.01 vs. model group.

To assess whether LXJD formula exerts therapeutic effects in a psoriasis mouse model, we applied IMQ on the shaved back skin of the animals for seven consecutive days. Subsequently, the skin developed typical erythema, scaling, and thickening. Consistent with the systems pharmacology analysis, mice treated with LXJD formula had smoother skin, less erythema, and sparser scales (Figure 5A). H&E staining of the skin lesion in the model group showed increased epidermal thickening, parakeratosis, and residual condensation of nuclei in the stratum corneum, representing typical characteristics of psoriasis-like skin lesions. These pathological features were ameliorated by LXJD formula (Figures 5B,F).

Loricrin is a keratinocyte differentiation marker that is mainly expressed in the cytoplasm and distributed throughout the stratum corneum of normal mouse skin. In the model group, low levels of loricrin indicated abnormal keratinocyte differentiation, which was reversed in animals treated with LXJD (*p* < 0.01) (Figures 5D,H). The expression of Ki-67, a marker strongly associated with cell proliferation and immune dysregulation (Alhosaini et al., 2021), was dramatically decreased in the LXJD-treated groups (*p* < 0.01) (Figures 5E,I), indicating that the IMQ-induced uncontrolled proliferation of basal keratinocytes was ameliorated.

### LiangXueJieDu Formula Inhibited T Cell Infiltration in an Establishment of Imiquimod-Induced Psoriasis-Like Mouse Model

We further examined the effect of LXJD formula on CD3+ T cell infiltration in the dermis. The control mice showed fewer CD3+ T cells in the dermis. The T cell infiltration in the dermis and epidermis was markedly higher in the model group than in the control group. On the other hand, LXJD and MTX groups showed reduced CD3+ T cell infiltration in the skin (*p* < 0.01) (Figures 5C,G).

### LiangXueJieDu Formula Regulated the Expression of Inflammatory Cytokines and Factors in an Establishment of Imiquimod-Induced Psoriasis-Like Mouse Model

**FIGURE 6:**
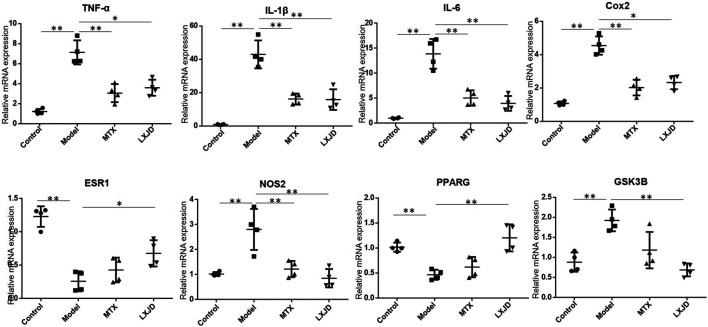
The mRNA expression of key inflammatory cytokines and predicted genes in the skin lesion of the psoriasis-like mouse model. Values were normalized to β-actin and expressed relative to the control group. Data are shown as mean ± SD. n = 4 mice. *p < 0.05 and **p < 0.01 vs. model group.

We determined the mRNA expression of several inflammatory cytokines and factors in the skin lesions and found that the levels of *Il1b*, *Il6*, *Tnfa*, *Cox2*, *Nos2*, and *Gsk3b* were significantly increased, while those of *Esr1* and *Pparg* mRNAs were suppressed in the lesions of the model group. Moreover, compared to the model group, the expression of *Il1b*, *Il6*, *Tnfa*, *Cox2*, *Nos2,* and *Gsk3b* mRNA declined in the LXJD group (*p* < 0.01 or *p* < 0.05), and that of *Esr1* and *Pparg* mRNAs reversed after LXJD formula administration (*p* < 0.01 or *p* < 0.05) (Figure 6).

### LiangXueJieDu Formula Improved the Establishment of Imiquimod-Induced Psoriasis-Like Dysfunction Pathway

**FIGURE 7:**
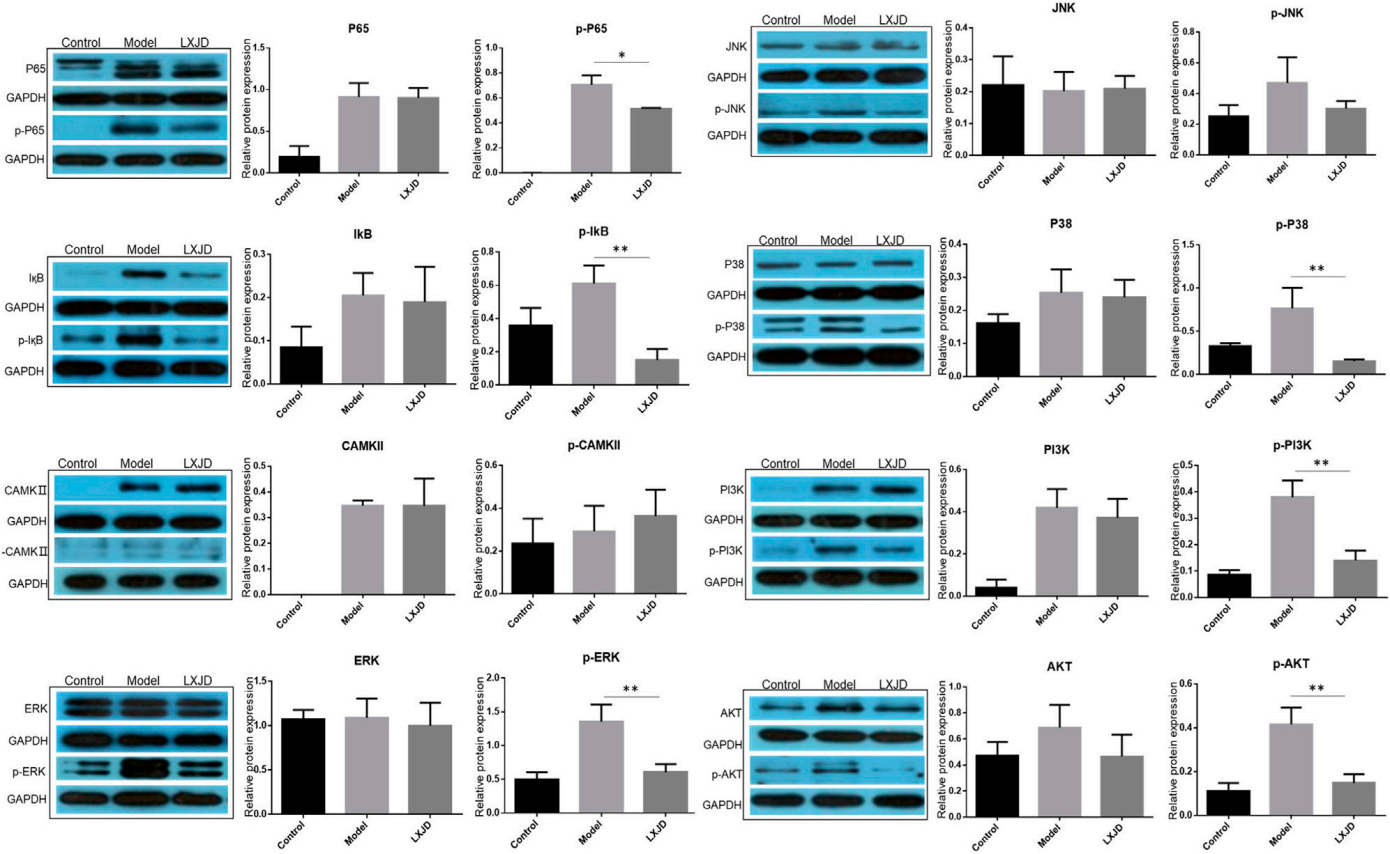
Effects of LXJD formula on IMQ-induced psoriasis-like dysfunction pathway. Levels of P65, p-P65, IқB, p-IқB, CAMKⅡ, p-CAMKⅡ, ERK, p-ERK, P38, p-P38, JNK, p-JNK, PI3K, p-PI3K, AKT, and p-AKT proteins in skin lesions are detected by Western blot. Semiquantitative analysis was performed. *p < 0.05, **p < 0.01 indicating significant inhibition of LXJD as compared to model group. Data are shown as mean ± SD. n = 3 mice.

To further validate the prediction of systematic pharmacological analysis, we examined the effect of LXJD formula on key proteins in integrated psoriasis-related pathways, including MAPK, calcium, PI3K/AKT, and NF-қB signaling pathways using Western blot. IMQ application significantly increased the level of p-P65, p-IқB, p-ERK, p-P38, p-JNK, p-PI3K, p-AKT, and p-CAMKⅡ in skin lesions. Compared to the model group, LXJD formula treatment remarkably inhibited the phosphorylation of p-P65, p-IқB, p-ERK, p-P38, p-PI3K, and p-AKT (*p* < 0.01 or *p* < 0.05), but showed a negligible inhibitory effect on p-JNK and p-CAMKⅡ phosphorylation, indicating that LXJD formula exerts its therapeutic effect by inhibiting the MAPK, PI3K/AKT, and NF-қB signaling pathways to a large extent (Figure 7).

## Discussion

TCM has been used in clinical treatment in China for more than 2000 years and has accumulated a wealth of experience in the treatment of psoriasis. Our previous study has shown that the administration of LXJD formula in psoriasis vulgaris patients with blood-heat syndrome was associated with significantly better outcomes and a lower recurrence rate compared to standard Western medicine (cetirizine hydrochloride, vitamin C, and vitamin B complex) (Sun et al., 2020). However, bioactive ingredients with potential anti-psoriatic activities and specific targets have not yet been identified. TCM is a complex material system encompassing multiple active compounds, multiple targets of action, and related diseases; while it does not correspond to the theory of “single gene, single target, and single disease” (Lv et al., 2019). Therefore, exploring the targets and mechanism of LXJD formula in the treatment of psoriasis through a systems pharmacology method combined with a screening of active compounds, drug targets, and network analysis is essential.

In this study, we identified 144 compounds in the active fractions of LXJD formula using an *in silico* integrative model-ADME system with the following thresholds: OB ≥ 30%, DL ≥ 0.18, Caco-2 > -0.4, and AlogP <5. Subsequently, a final list of 125 potential targets for the 144 bioactive compounds was obtained by several integrated approaches, including the WES algorithm, TCMSP database, PharmGkb, Comparative Toxicogenomics database, and Therapeutic Targets database. These potential targets could be classified into the following subgroups: cytokines and chemokines, enzymes, signaling molecules, and transcription factors, and nuclear receptors. Most of these targets exhibited abnormal expression and were designated as the therapeutic targets in psoriasis, such as the cytokines and chemokines (IL-1β, IL-6, TNF, IFN-γ, CXCL8, CCL2). As expected, drugs targeting TNF-α have been applied in the clinic and have shown promising therapeutic effects on psoriasis (Chima and Lebwohl, 2018). Moreover, a total of 125 potential targets were significantly enriched as GO terms (BPs), including inflammatory response, cell proliferation, inflammatory response, NF-κB transcription factor activity, and cytokine production, which are strongly associated with the pathogenesis of psoriasis. Then, the compound-target network was constructed, showing complex interactions between 144 active compounds and 125 targets. Among these, ESR1 was the most common target of 113 compounds, constituting up to 77.1% of the total candidates. Reportedly, *ESR1* is the top-ranked hub gene, which is downregulated in psoriasis and related to anti-apoptotic functions (Zeng et al., 2016), deeming it as a potential therapeutic target for psoriasis. Consistently, our compound-target network indicated that the active compounds from LXJD formula could activate ESR1. Moreover, *IL1B* and *STAT3* were also reported as hub genes in psoriasis (Choudhary et al., 2020) and targets of LXJD formula. In this compound-target network, most of the active compounds were connected to multiple targets and exerted pharmacological effects. For example, quercetin exhibited the highest number of target interactions. Quercetin possesses anti-tumor activity (Xu et al., 2015) and anti-proliferative effects (Delgado et al., 2014); also, it is shown to have appreciable anti-psoriasis effects in IMQ-induced mice. The underlying mechanism might involve the improvement of antioxidant, anti-inflammatory activity, and inhibition on NF-κB signaling pathway activation (Chen et al., 2017). In addition, the anti-psoriasis effects of Paeonol (Meng et al., 2017), Baicalin (Hung et al., 2018), Paeoniflorin (Zhao et al., 2016), and Wogonin (Chi et al., 2003) have been demonstrated in an IMQ-induced psoriasis-like mouse model.

To further elucidate the complex mechanism of LXJD formula in the treatment of psoriasis, we established an integrated map of “psoriasis-related pathways” by integrating the key pathways obtained from the KEGG database using target-pathway network analysis. This map showed that the active compounds in LXJD formula interacting with these predicted targets are associated with the MAPK, calcium, PI3K/AKT, and NF-κB signaling pathways. These interactions regulate inflammation, cell proliferation, metabolism, cell survival, cell cycle, and immune response, and ultimately improve psoriasis.

Different complex diseases caused by the same disease gene exhibit overlapping protein-protein interactions and biological processes. Therefore, when a drug acts on the proteins associated with multiple diseases, it might show multiple therapeutic effects (Li et al., 2012). For example, peroxisome proliferator-activated receptor gamma (PPARG or PPAR-γ), a nuclear receptor, is one of the primary targets of LXJD formula, according to our compound-target network. PPAR-γ can bind to DNA and regulate the transcription of genes involved in lipid and glucose metabolism. Activation of PPAR-γ has favorable effects on adipocyte function, insulin sensitivity, lipoprotein metabolism, and vascular structure and function. PPAR-γ has been recognized as a therapeutic target in cardiovascular disease and diabetes mellitus. In addition, several studies demonstrated that PPAR-γ agonists may have antidepressant properties, open-label administration of the PPAR-γ agonist, pioglitazone, was associated with improvement in depressive symptoms and reduced cardiometabolic risk (Kemp et al., 2014). In the skin, PPAR-γ has a critical role in keratinocyte homeostasis, exhibiting pro-differentiation, antiproliferation, and immunomodulatory functions. The expression of PPAR-γ was reduced in a psoriasis hyperproliferative epidermis (Lima et al., 2013). Reportedly, diabetic patients treated with troglitazone (PPAR-γ agonist) showed substantial improvement in their psoriatic plaques (Pershadsingh et al., 1998). In addition to relieving the symptoms of patients with chronic psoriasis, this drug has modified the abnormal phenotype of transplanted psoriatic skin (Hanley et al., 1999). Furthermore, treatment with thiazolidinediones (PPAR-γ agonist) in patients with plaque psoriasis improved their cutaneous symptoms (Pershadsingh et al., 1998; Ellis et al., 2000). Based on the target-disease network, we could speculate that LXJD formula has potential pharmacological effects on immune system disease, endocrine system disease, skin disease, cardiovascular disease, metabolic disease, and mental disorder. Consistently, a previous study identified that psoriasis is not confined to skin impairment, and an increased morbidity of metabolic syndrome, cardiovascular disease, depression, and anxiety are also associated complications (Lima et al., 2013). Chronic inflammation caused by disorganized immune activation and correlated systemic abnormalities might underpin the common pathogenesis of psoriasis and related diseases. There is evidence that an imbalance between different T cells, e.g., T helper cells 17 (Th17) vs. regulatory T cells (Treg) and abnormal cytokine production are the driving forces in common for the progression and development of mental disorder and immune system disease (Ahmad et al., 2017; Ahmad et al., 2017; Bakheet et al., 2019; Ahmad et al., 2020). Many compounds, which have been shown to have promising effects in multiple immune-mediated inflammatory disorders such as asthma, rheumatoid arthritis, and alcoholic liver disease, have therapeutic potential in psoriatic inflammation as well (Alzahrani et al., 2019; Al-Harbi et al., 2020; Nadeem et al., 2020). Collectively, the herbs in LXJD formula exhibit efficacy in psoriasis treatment and in treating related complications, indicating that multiple diseases may be treated using a common herbal medicine.

Metabolomics data might provide useful information to explore the mechanisms underlying these associated diseases, especially metabolism disorders (Lee et al., 2003). Herein, we employed LC-MS to analyze psoriasis patients’ serum metabolomics and investigate the metabolic changes associated with LXJD formula treatment. In concordance with the previous study, the elements of glycerophospholipid metabolism were significantly altered in the plasma of psoriatic patients (Zeng et al., 2017). The current metabolomics results indicated that glycerophospholipid metabolism involving 11 metabolites was significantly influenced in psoriasis patients according to MetPA, and the most influencing targets were related to the therapeutic intervention of LXJD formula against psoriasis. The compound-target network showed that PPAR-γ could be activated by LXJD formula. There is evidence that the PPAR-γ agonist could regulate glycerophospholipid metabolism (Tan et al., 2017), which is consistent with our metabolomics data showing that LXJD formula improved the perturbed metabolic pathways of glycerophospholipid metabolism. Based on this, we inferred that LXJD formula might regulate the glycerophospholipid metabolism through activating PPAR-γ. In addition, abnormalities in glycerophospholipid metabolism are the characteristics of diabetes mellitus (Xu et al., 2013). Dang et al. also revealed that the alteration of glycerophospholipid metabolism pathways is associated with atherosclerosis (Dang et al., 2016). Therefore, glycerophospholipid metabolism might be a drug target for psoriasis and related complications, including diabetes mellitus and atherosclerosis. LXJD formula could treat these associated diseases by regulating the altered metabolism.

Finally, we utilized an IMQ-induced psoriasis-like mouse model to validate the predicted targets and mechanisms of LXJD formula. In line with the current findings from systems pharmacology, LXJD formula could ameliorate psoriasis lesions, inhibit keratinocytes proliferation, improve keratinocytes differentiation, and suppress the infiltration of CD3+ T cells in an IMQ-induced psoriasis-like mouse model. This therapeutic effect was mediated by inhibiting the MAPK, PI3K/AKT, and NF-қB signaling pathways and reducing the subsequent release of inflammatory cytokines.

## Conclusion and Limitation

In conclusion, our systems pharmacological network identified active compounds and potential target proteins and elucidated the pharmacological mechanism of LXJD formula for the treatment of psoriasis and related diseases. These targets have been validated by our *in vivo* experiments. The metabolic changes associated with LXJD formula treatment adequately complemented the interaction between the Chinese herbal compounds and body metabolism, thereby revealing the underlying mechanism. These studies provided convincing evidence and a biological basis for LXJD formula in the treatment of psoriasis, contributing to its clinical application and generalization.

Certainly, there are still some limitations in our study. Even though LXJD formula has been proven as a safe treatment for psoriasis (Sun et al., 2020) and been used widely in China for decades, it still cause concerns about its toxicity due to different sources of herbs and different types of concoctions. In this animal study, no significant impact on body weight was found after LXJD formula treatment (data not shown). Nonetheless, further toxicity/safety evaluation studies need to be done in future. Components of Chinese herbal medicine are complicated, it is not clarified whether there are new intermediate metabolites or side effects caused by the interaction between compounds. We were not able to validate all of the 144 compounds in this formula by UPLC-MS analysis. Besides, a new intermediate metabolite might have an effect on psoriasis in a pre-clinical *in vivo* psoriasis model and/or human trial testing as well. We investigated the whole formula effect on a psoriasis-like animal model and corresponding predicted targets, but the effect of individual compounds on corresponding predicted targets remains unclear. Despite these potentially interesting associations, cautious interpretation and further validation tests are warranted.

Although further validation tests will be required to support deep assessments, the current study combining systems pharmacology, metabolomics, and experiment evaluation illustrated the correlation among active compounds, targets, and metabolism, contributing to the understanding of the mechanism of LXJD formula in the treatment of psoriasis.

## Data Availability

The original contributions presented in the study are included in the article/Supplementary Material, further inquiries can be directed to the corresponding author.
