# Supplementary material for: Systems Pharmacology Approach and Experiment Evaluation Reveal Multidimensional Treatment Strategy of LiangXueJieDu Formula for Psoriasis
Source: Front Pharmacol. 2021 Jun 8;12:626267. doi: 10.3389/fphar.2021.626267 (PMC8217833; doi:10.3389/fphar.2021.626267)
Supplement: Supplementary file 1 [file Table1.DOCX]

Table S1 Bioactive compounds identification for each herb in LXJD formula

| **NO.** | **Mol ID** | **Compound** | **Herb** | **Satisfy the screening model** | **Degree** | **Structure** |
| --- | --- | --- | --- | --- | --- | --- |
| 1 | mol01 | palmitic acid | Isatidis Radix | NO | 11 |  |
| 2 | mol02 | beta-sitosterol | Isatidis Radix  Imperatae Rhizoma | NO | 17 |  |
| 3 | mol03 | sitosterol | Isatidis Radix  Lithospermum Erythrorhizon | NO | 6 |  |
| 4 | mol04 | Stigmasterol | Isatidis Radix  Imperatae Rhizoma | NO | 17 |  |
| 5 | mol05 | cholesterol | Isatidis Radix | Yes | 1 |  |
| 6 | mol06 | acacetin | Isatidis Radix | Yes | 17 |  |
| 7 | mol07 | Isaindigodione | Isatidis Radix | Yes | 5 |  |
| 8 | mol08 | 2-O-beta-D-glucopyranosyl-2H-1,4-benzoxazin-3(4H)-one | Isatidis Radix | Yes | 6 |  |
| 9 | mol09 | Eupatorin | Isatidis Radix | Yes | 7 |  |
| 10 | mol10 | 3-[[(2R,3R,5R,6S)-3,5-dihydroxy-6-(1H-indol-3-yloxy)-4-oxooxan-2-yl]methoxy]-3-oxopropanoic acid | Isatidis Radix | Yes | 6 |  |
| 11 | mol11 | Dinatin | Isatidis Radix | Yes | 8 |  |
| 12 | mol12 | (-)-taxifolin | Isatidis Radix | Yes | 7 |  |
| 13 | mol13 | bis[(2R)-2-ethylhexyl] benzene-1,2-dicarboxylate | Isatidis Radix | Yes | 1 |  |
| 14 | mol14 | 24-Ethylcholest-4-en-3-one | Isatidis Radix | Yes | 1 |  |
| 15 | mol15 | quindoline | Isatidis Radix | Yes | 2 |  |
| 16 | mol16 | hydroxyindirubin | Isatidis Radix | Yes | 7 |  |
| 17 | mol17 | poriferast-5-en-3beta-ol | Isatidis Radix | Yes | 1 |  |
| 18 | mol18 | Ineketone | Isatidis Radix | Yes | 1 |  |
| 19 | mol19 | Sinoacutine | Isatidis Radix | Yes | 7 |  |
| 20 | mol20 | Indigo | Isatidis Radix | Yes | 1 |  |
| 21 | mol21 | (2Z)-2-(2-oxoindolin-3-ylidene)indolin-3-one | Isatidis Radix | Yes | 5 |  |
| 22 | mol22 | 2-(9-((3-methyl-2-oxopent-3-en-1-yl)oxy)-2-oxo-1,2,8,9-tetrahydrofuro[2,3-h]quinolin-8-yl)propan-2-yl acetate | Isatidis Radix | Yes | 6 |  |
| 23 | mol23 | adenosine | Isatidis Radix | NO | 6 |  |
| 24 | mol24 | Liquiritigenin | Isatidis Radix | Yes | 8 |  |
| 25 | mol25 | (E)-2-[(3-indole)cyanomethylene-]-3-indolinone | Isatidis Radix | Yes | 5 |  |
| 26 | mol26 | neohesperidin_qt | Isatidis Radix | Yes | 7 |  |
| 27 | mol27 | rosasterol | Isatidis Radix | Yes | 1 |  |
| 28 | mol28 | Sinensetin | Isatidis Radix | Yes | 7 |  |
| 29 | mol29 | Stigmasta-5,22-diene-3beta,7alpha-diol | Isatidis Radix | Yes | 1 |  |
| 30 | mol30 | Stigmasta-5,22-diene-3beta,7beta-diol | Isatidis Radix | Yes | 1 |  |
| 31 | mol31 | 6-(3-oxoindolin-2-ylidene)indolo[2,1-b]quinazolin-12-one | Isatidis Radix |  | 2 |  |
| 32 | mol32 | (E)-3-(3,5-dimethoxy-4-hydroxy-benzylidene)-2-indolinone | Isatidis Radix | Yes | 7 |  |
| 33 | mol33 | (E)-3-(3,5-dimethoxy-4-hydroxyb-enzylidene)-2-indolinone | Isatidis Radix | Yes | 8 |  |
| 34 | mol34 | Indican, plant | Isatidis Radix | NO | 5 |  |
| 35 | mol35 | Isaindigotone | Isatidis Radix | Yes | 7 |  |
| 36 | mol36 | Glucobrassicin-1-Sulfonate_qt | Isatidis Radix | Yes | 2 |  |
| 37 | mol37 | isovitexin | Isatidis Radix | Yes | 6 |  |
| 38 | mol38 | Bifendate | Imperatae Rhizoma | Yes | 6 |  |
| 39 | mol39 | Friedelin | Imperatae Rhizoma | NO | 1 |  |
| 40 | mol40 | fernenol | Imperatae Rhizoma | NO | 1 |  |
| 41 | mol41 | Luteolinidin | Imperatae Rhizoma | Yes | 6 |  |
| 42 | mol42 | simiarenol | Imperatae Rhizoma | NO | 1 |  |
| 43 | mol43 | isoarborinol | Imperatae Rhizoma | NO | 1 |  |
| 44 | mol44 | isochlorogenic,acid | Imperatae Rhizoma | NO | 1 |  |
| 45 | mol45 | Coixol | Imperatae Rhizoma | Yes | 3 |  |
| 46 | mol46 | Mandenol | Lithospermum Erythrorhizon  Saposhnikoviae Radix | Yes | 3 |  |
| 47 | mol47 | Ethyl oleate (NF) | Lithospermum Erythrorhizon  Radix Paeoniae Rubra | Yes | 2 |  |
| 48 | mol48 | 1-methoxyacetylshikonin | Lithospermum Erythrorhizon | Yes | 9 |  |
| 49 | mol49 | Propionylshikonin | Lithospermum Erythrorhizon | Yes | 6 |  |
| 50 | mol50 | acetylshikonin | Lithospermum Erythrorhizon | Yes | 8 |  |
| 51 | mol51 | Arnebin 7 | Lithospermum Erythrorhizon | Yes | 7 |  |
| 52 | mol52 | Isoarnebin 4 | Lithospermum Erythrorhizon | Yes | 6 |  |
| 53 | mol53 | lithospermidin A | Lithospermum Erythrorhizon | Yes | 4 |  |
| 54 | mol54 | shikonofuran C | Lithospermum Erythrorhizon | NO | 6 |  |
| 55 | mol55 | shikonofuran B | Lithospermum Erythrorhizon | NO | 6 |  |
| 56 | mol56 | arnebinol | Lithospermum Erythrorhizon | Yes | 5 |  |
| 57 | mol57 | arnebinone | Lithospermum Erythrorhizon | NO | 4 |  |
| 58 | mol58 | 5-[(E)-5-(3-furyl)-2-methyl-pent-2-enyl]-2,3-dimethoxy-p-benzoquinone | Lithospermum Erythrorhizon | Yes | 5 |  |
| 59 | mol59 | Lithospermidin B | Lithospermum Erythrorhizon | Yes | 3 |  |
| 60 | mol60 | quercetin | Sophora Japonica L. | Yes | 79 |  |
| 61 | mol61 | isorhamnetin | Sophora Japonica L. | Yes | 12 |  |
| 62 | mol62 | kaempferol | Sophora Japonica L.  Cortex Moutan | Yes | 33 |  |
| 63 | mol63 | soyasponin I | Sophora Japonica L. | NO | 7 |  |
| 64 | mol64 | N-[6-(9-acridinylamino)hexyl]benzamide | Sophora Japonica L. | Yes | 2 |  |
| 65 | mol65 | kaikasaponinIII_qt | Sophora Japonica L. | NO | 1 |  |
| 66 | mol66 | quercetin-3'-methyl ether | Sophora Japonica L. | Yes | 1 |  |
| 67 | mol67 | quercetin | Cortex Moutan | Yes | 1 |  |
| 68 | mol68 | Mairin | Cortex Moutan  Radix Paeoniae Rubra | Yes | 8 |  |
| 69 | mol69 | sitosterol | Cortex Moutan  Radix Paeoniae Rubra | Yes | 16 |  |
| 70 | mol70 | kaempferol | Cortex Moutan | Yes | 5 |  |
| 71 | mol71 | (+)-catechin | Cortex Moutan | Yes | 4 |  |
| 72 | mol72 | paeonol | Cortex Moutan | NO | 2 |  |
| 73 | mol73 | Paeonolide | Cortex Moutan | NO | 1 |  |
| 74 | mol74 | paeonoside | Cortex Moutan  Radix Paeoniae Rubra | NO | 1 |  |
| 75 | mol75 | trametenolic acid | Smilacis Glabrae Rhixoma | NO | 1 |  |
| 76 | mol76 | Cerevisterol | Smilacis Glabrae Rhixoma | Yes | 1 |  |
| 77 | mol77 | ergosta-7,22E-dien-3beta-ol | Smilacis Glabrae Rhixoma | Yes | 1 |  |
| 78 | mol78 | Ergosterol peroxide | Smilacis Glabrae Rhixoma | Yes | 1 |  |
| 79 | mol79 | hederagenin | Smilacis Glabrae Rhixoma | Yes | 6 |  |
| 80 | mol80 | wogonin | Saposhnikoviae Radix  Dictamni Cortex | Yes | 21 |  |
| 81 | mol81 | Ammidin | Saposhnikoviae Radix | Yes | 5 |  |
| 82 | mol82 | isoimperatorin | Saposhnikoviae Radix | Yes | 3 |  |
| 83 | mol83 | Marmesin | Saposhnikoviae Radix | Yes | 8 |  |
| 84 | mol84 | Phellopterin | Saposhnikoviae Radix | Yes | 9 |  |
| 85 | mol85 | Prangenidin | Saposhnikoviae Radix | Yes | 5 |  |
| 86 | mol86 | methyl icosa-11,14-dienoate | Saposhnikoviae Radix | Yes | 3 |  |
| 87 | mol87 | heptadeca-1,8-dien-4,6-diyn-3,10-diol | Saposhnikoviae Radix | Yes | 2 |  |
| 88 | mol88 | 11-hydroxy-sec-o-beta-d-glucosylhamaudol_qt | Saposhnikoviae Radix | Yes | 5 |  |
| 89 | mol89 | 3'-O-Acetylhamaudol | Saposhnikoviae Radix | NO | 6 |  |
| 90 | mol90 | anomalin | Saposhnikoviae Radix | Yes | 3 |  |
| 91 | mol91 | divaricatacid | Saposhnikoviae Radix | Yes | 4 |  |
| 92 | mol92 | divaricatol | Saposhnikoviae Radix | Yes | 6 |  |
| 93 | mol93 | ledebouriellol | Saposhnikoviae Radix | Yes | 4 |  |
| 94 | mol94 | phelloptorin | Saposhnikoviae Radix | Yes | 6 |  |
| 95 | mol95 | 5-O-Methylvisamminol | Saposhnikoviae Radix | Yes | 7 |  |
| 96 | mol96 | Decursin | Saposhnikoviae Radix | Yes | 8 |  |
| 97 | mol97 | lignoceric acid | Saposhnikoviae Radix | NO | 2 |  |
| 98 | mol98 | catapol_qt | Rehmanniae Radix Praeparata | Yes | 1 |  |
| 99 | mol99 | Rehmaionoside C | Rehmanniae Radix Praeparata | NO | 1 |  |
| 100 | mol100 | catalpol | Rehmanniae Radix Praeparata | NO | 4 |  |
| 101 | mol101 | Epi-Friedelanol | Radix Paeoniae Rubra | NO | 1 |  |
| 102 | mol102 | 3,4,5-trihydroxybenzoic acid | Radix Paeoniae Rubra | Yes | 11 |  |
| 103 | mol103 | ellagic acid | Radix Paeoniae Rubra | Yes | 13 |  |
| 104 | mol104 | baicalein | Radix Paeoniae Rubra | Yes | 21 |  |
| 105 | mol105 | Baicalin | Radix Paeoniae Rubra | Yes | 2 |  |
| 106 | mol106 | Spinasterol | Radix Paeoniae Rubra | Yes | 1 |  |
| 107 | mol107 | campest-5-en-3beta-ol | Radix Paeoniae Rubra  Dictamni Cortex | Yes | 2 |  |
| 108 | mol108 | (2R,3R)-4-methoxyl-distylin | Radix Paeoniae Rubra | Yes | 7 |  |
| 109 | mol109 | stigmast-7-en-3-ol | Radix Paeoniae Rubra | Yes | 1 |  |
| 110 | mol110 | paeonin,a | Radix Paeoniae Rubra | NO | 2 |  |
| 111 | mol111 | paeonin,a_qt | Radix Paeoniae Rubra | Yes | 3 |  |
| 112 | mol112 | paeonin,b | Radix Paeoniae Rubra | NO | 1 |  |
| 113 | mol113 | paeonin,b_qt | Radix Paeoniae Rubra | Yes | 3 |  |
| 114 | mol114 | paeoniflorin | Radix Paeoniae Rubra | Yes | 3 |  |
| 115 | mol115 | luteolin | Dictamni Cortex | Yes | 34 |  |
| 116 | mol116 | Skimmianin | Dictamni Cortex | Yes | 5 |  |
| 117 | mol117 | 3'-O-methyl taxifolin | Dictamni Cortex | Yes | 7 |  |
| 118 | mol118 | Dasycarpamin | Dictamni Cortex | Yes | 12 |  |
| 119 | mol119 | O-ethylnor-γ-fagarine | Dictamni Cortex | Yes | 2 |  |
| 120 | mol120 | Dictamdiol A | Dictamni Cortex | Yes | 4 |  |
| 121 | mol121 | preskimmianine | Dictamni Cortex | Yes | 11 |  |
| 122 | mol122 | tirucallane | Dictamni Cortex | NO | 1 |  |
| 123 | mol123 | 9alpha-hydroxyfraxinellone-9-o-beta-d-glucoside | Dictamni Cortex | Yes | 1 |  |
| 124 | mol124 | psoralen | Dictamni Cortex | Yes | 1 |  |
| 125 | mol125 | ursolic acid | Hedyotis Diffusae Herba | NO | 35 |  |
| 126 | mol126 | p-coumaric acid | Hedyotis Diffusae Herba | Yes | 4 |  |
| 127 | mol127 | 2-hydroxy-3-methylanthraquinone | Hedyotis Diffusae Herba | NO | 17 |  |
| 128 | mol128 | E-6-O-p-methoxycinnamoyl scandoside methyl ester_qt | Hedyotis Diffusae Herba | NO | 7 |  |
| 129 | mol129 | Oleanolic acid-28-O-beta-D-glucopyranoside | Hedyotis Diffusae Herba | NO | 1 |  |
| 130 | mol130 | scandoside_qt | Hedyotis Diffusae Herba | Yes | 3 |  |
| 131 | mol131 | Poriferasterol | Hedyotis Diffusae Herba | Yes | 1 |  |
| 132 | mol132 | scandoside_qt | Hedyotis Diffusae Herba | NO | 4 |  |
| 133 | mol133 | 3-Epioleanolic acid | Hedyotis Diffusae Herba | Yes | 1 |  |
| 134 | mol134 | 2-methoxy-3-methyl-9,10-anthraquinone | Hedyotis Diffusae Herba | Yes | 15 |  |
| 135 | mol135 | rutin | Hedyotis Diffusae Herba | NO | 15 |  |
| 136 | mol136 | POlyPhyllin 1 | Bistortae Rhizoma | NO | 7 |  |
| 137 | mol137 | Daucosterol | Bistortae Rhizoma | NO | 7 |  |
| 138 | mol138 | Daucosterol | Bistortae Rhizoma | Yes | 4 |  |
| 139 | mol139 | Creatinine | Bistortae Rhizoma | Yes | 1 |  |
| 140 | mol140 | POlyPhyllin 3 | Bistortae Rhizoma | Yes | 8 |  |
| 141 | mol141 | α- Ecdysterone | Bistortae Rhizoma | NO | 4 |  |
| 142 | mol142 | β- Ecdysterone | Bistortae Rhizoma | NO | 4 |  |
| 143 | mol143 | methylprotodioscin | Bistortae Rhizoma | NO | 6 |  |
| 144 | mol144 | Methylprotodioscin_qt | Bistortae Rhizoma | NO | 7 |  |
