# Supplementary material for: Systems Pharmacology Approach and Experiment Evaluation Reveal Multidimensional Treatment Strategy of LiangXueJieDu Formula for Psoriasis
Source: Front Pharmacol. 2021 Jun 8;12:626267. doi: 10.3389/fphar.2021.626267 (PMC8217833; doi:10.3389/fphar.2021.626267)
Supplement: Supplementary file 2 [file Table2.DOCX]

**Supp Table S2 The information of targets**

| **NO.** | **Target-name** | **Gene-Name** | **Degree** |
| --- | --- | --- | --- |
| 1 | Estrogen receptor | ESR1 | 113 |
| 2 | Prostaglandin G/H synthase 2 | PTGS2 | 86 |
| 3 | Nitric oxide synthase, inducible | NOS2 | 81 |
| 4 | Peroxisome proliferator activated receptor gamma | PPARG | 60 |
| 5 | Glycogen synthase kinase-3 beta | GSK3B | 56 |
| 6 | Heat shock protein HSP 90 | HSP90AA1 | 55 |
| 7 | mRNA of Protein-tyrosine phosphatase, non-receptor type 1 | PTPN1 | 44 |
| 8 | Nitric-oxide synthase, endothelial | NOS3 | 33 |
| 9 | Beta-2 adrenergic receptor | ADRB2 | 20 |
| 10 | Transcription factor p65 | RELA | 13 |
| 11 | Caspase-3 | CASP3 | 12 |
| 12 | Muscarinic acetylcholine receptor M2 | CHRM2 | 10 |
| 13 | Apoptosis regulator Bcl-2 | BCL2 | 10 |
| 14 | Tumor necrosis factor | TNF | 10 |
| 15 | Glucocorticoid receptor | NR3C1 | 10 |
| 16 | Corticosteroid 11-beta-dehydrogenase isozyme 2 | HSD11B2 | 10 |
| 17 | Glucose-6-phosphate 1-dehydrogenase | G6PD | 10 |
| 18 | Apoptosis regulator BAX | BAX | 8 |
| 19 | Mu-type opioid receptor | OPRM1 | 7 |
| 20 | Sodium-dependent dopamine transporter | SLC6A3 | 7 |
| 21 | Cellular tumor antigen p53 | TP53 | 7 |
| 22 | Cyclin-dependent kinase inhibitor 1 | CDKN1A | 6 |
| 23 | RAC-alpha serine/threonine-protein kinase | AKT1 | 6 |
| 24 | Interleukin-6 | IL6 | 6 |
| 25 | Interstitial collagenase | MMP1 | 5 |
| 26 | Intercellular adhesion molecule 1 | ICAM1 | 5 |
| 27 | Glutathione S-transferase P | GSTP1 | 5 |
| 28 | Vascular endothelial growth factor A | VEGFA | 5 |
| 29 | Matrix metalloproteinase-9 | MMP9 | 5 |
| 30 | NF-kappa-B inhibitor alpha | NFKBIA | 5 |
| 31 | Amyloid beta A4 protein | APP | 5 |
| 32 | 5-hydroxytryptamine 2A receptor | HTR2A | 4 |
| 33 | Caspase-8 | CASP8 | 4 |
| 34 | 72 kDa type IV collagenase | MMP2 | 4 |
| 35 | Interleukin-8 | CXCL8 | 4 |
| 36 | Calcitonin gene-related peptide type 1 receptor | CALCRL | 4 |
| 37 | Neurotensin receptor type 1 | NTSR1 | 4 |
| 38 | Peptidyl-prolyl cis-trans isomerase FKBP5 | FKBP5 | 4 |
| 39 | Beta-galactosidase | GLB1 | 4 |
| 40 | Aldose reductase | AKR1B1 | 3 |
| 41 | Leukotriene A-4 hydrolase | LTA4H | 3 |
| 42 | Amine oxidase [flavin-containing] A | MAOA | 3 |
| 43 | Calcium-activated potassium channel subunit alpha 1 | KCNMA1 | 3 |
| 44 | Interleukin-10 | IL10 | 3 |
| 45 | Phosphatidylinositol-3,4,5-trisphosphate 3-phosphatase and dual-specificity protein phosphatase PTEN | PTEN | 3 |
| 46 | Fatty acid synthase | FASN | 3 |
| 47 | Tumor necrosis factor ligand superfamily member 6 | FASLG | 3 |
| 48 | Heme oxygenase 1 | HMOX1 | 3 |
| 49 | E-selectin | SELE | 3 |
| 50 | Arachidonate 5-lipoxygenase | ALOX5 | 3 |
| 51 | Aryl hydrocarbon receptor | AHR | 3 |
| 52 | Solute carrier family 2, facilitated glucose transporter member 4 | SLC2A4 | 3 |
| 53 | Insulin receptor | INSR | 3 |
| 54 | Glutathione S-transferase Mu 1 | GSTM1 | 3 |
| 55 | Neutrophil cytosol factor 1 | NCF1 | 3 |
| 56 | Bcl-2-like protein 1 | BCL2L1 | 3 |
| 57 | Mitogen-activated protein kinase 1 | MAPK1 | 3 |
| 58 | Superoxide dismutase [Cu-Zn] | SOD1 | 3 |
| 59 | Interleukin-1 beta | IL1B | 3 |
| 60 | Protein kinase C beta type | PRKCB | 3 |
| 61 | Interleukin-2 | IL2 | 3 |
| 62 | Insulin-like growth factor II | IGF2 | 3 |
| 63 | C-C motif chemokine 5 | CCL5 | 3 |
| 64 | Cathepsin D | CTSD | 2 |
| 65 | Alcohol dehydrogenase 1B | ADH1B | 2 |
| 66 | Beta-1 adrenergic receptor | ADRB1 | 2 |
| 67 | Transforming growth factor beta-1 | TGFB1 | 2 |
| 68 | Serum paraoxonase/arylesterase 1 | PON1 | 2 |
| 69 | Stromelysin-1 | MMP3 | 2 |
| 70 | Peroxisome proliferator-activated receptor delta | PPARD | 2 |
| 71 | Mitogen-activated protein kinase 8 | MAPK8 | 2 |
| 72 | Signal transducer and activator of transcription 1-alpha/beta | STAT1 | 2 |
| 73 | Cytochrome P450 1A2 | CYP1A2 | 2 |
| 74 | Vascular cell adhesion protein 1 | VCAM1 | 2 |
| 75 | Nuclear receptor subfamily 1 group I member 2 | NR1I2 | 2 |
| 76 | 26S proteasome non-ATPase regulatory subunit 3 | PSMD3 | 2 |
| 77 | Nuclear receptor subfamily 1 group I member 3 | NR1I3 | 2 |
| 78 | Epidermal growth factor receptor | EGFR | 2 |
| 79 | Hypoxia-inducible factor 1-alpha | HIF1A | 2 |
| 80 | C-C motif chemokine 2 | CCL2 | 2 |
| 81 | Interferon gamma | IFNG | 2 |
| 82 | Myeloperoxidase | MPO | 2 |
| 83 | Catalase | CAT | 2 |
| 84 | Histamine H1 receptor | HRH1 | 2 |
| 85 | 5-hydroxytryptamine 2C receptor | HTR2C | 2 |
| 86 | Adenosine A2a receptor | ADORA2A | 1 |
| 87 | Solute carrier family 22 member 5 | SLC22A5 | 1 |
| 88 | Aldo-keto reductase family 1 member C3 | AKR1C3 | 1 |
| 89 | Pro-epidermal growth factor | EGF | 1 |
| 90 | Ornithine decarboxylase | ODC1 | 1 |
| 91 | 78 kDa glucose-regulated protein | HSPA5 | 1 |
| 92 | Heat shock protein beta-1 | HSPB1 | 1 |
| 93 | Tissue-type plasminogen activator | PLAT | 1 |
| 94 | Plasminogen activator inhibitor 1 | SERPINE1 | 1 |
| 95 | Interleukin-1 alpha | IL1A | 1 |
| 96 | ATP-binding cassette sub-family G member 2 | ABCG2 | 1 |
| 97 | NAD(P)H dehydrogenase [quinone] 1 | NQO1 | 1 |
| 98 | Poly [ADP-ribose] polymerase 1 | PARP1 | 1 |
| 99 | C-X-C motif chemokine 2 | CXCL2 | 1 |
| 100 | Peroxisome proliferator-activated receptor alpha | PPARA | 1 |
| 101 | C-reactive protein | CRP | 1 |
| 102 | C-X-C motif chemokine 10 | CXCL10 | 1 |
| 103 | Osteopontin | SPP1 | 1 |
| 104 | Runt-related transcription factor 2 | RUNX2 | 1 |
| 105 | Insulin-like growth factor-binding protein 3 | IGFBP3 | 1 |
| 106 | Hexokinase-2 | HK2 | 1 |
| 107 | Fibronectin | FN1 | 1 |
| 108 | Lipopolysaccharide-binding protein | LBP | 1 |
| 109 | Cytochrome c | CYCS | 1 |
| 110 | Arachidonate 12-lipoxygenase, 12S-type | ALOX12 | 1 |
| 111 | E3 ubiquitin-protein ligase Mdm2 | MDM2 | 1 |
| 112 | Proliferating cell nuclear antigen | PCNA | 1 |
| 113 | Interleukin-4 | IL4 | 1 |
| 114 | Insulin | INS | 1 |
| 115 | Low affinity immunoglobulin epsilon Fc receptor | FCER2 | 1 |
| 116 | Integrin beta-2 | ITGB2 | 1 |
| 117 | Signal transducer and activator of transcription 3 | STAT3 | 1 |
| 118 | Cell division protein kinase 6 | CDK6 | 1 |
| 119 | Heparin-binding growth factor 2 | FGF2 | 1 |
| 120 | Stromelysin-2 | MMP10 | 1 |
| 121 | Granulocyte-macrophage colony-stimulating factor | CSF2 | 1 |
| 122 | Phosphatidylinositol-3,4,5-trisphosphate 5-phosphatase 2 | INPPL1 | 1 |
| 123 | G1/S-specific cyclin-D2 | CCND2 | 1 |
| 124 | Caspase-1 | CASP1 | 1 |
| 125 | Membrane primary amine oxidase | AOC3 | 1 |
