# Supplementary material for: Systems Pharmacology Approach and Experiment Evaluation Reveal Multidimensional Treatment Strategy of LiangXueJieDu Formula for Psoriasis
Source: Front Pharmacol. 2021 Jun 8;12:626267. doi: 10.3389/fphar.2021.626267 (PMC8217833; doi:10.3389/fphar.2021.626267)
Supplement: Supplementary file 3 [file Table3.DOCX]

**Supp Table S3 The relationship between compounds and targets**

| **NO.** | **Compound** | **Target** |
| --- | --- | --- |
| mol01 | palmitic acid | CTSD、ADH1B、NOS2、PPARG、PTGS2、NOS3、BCL2、IL10、TNF、PTEN、SLC22A5 |
| mol02 | beta-sitosterol | ESR1、NOS2、PPARG、PTGS2、PTPN1、GSK3B、HSP90AA1、HTR2A、CHRM2、ADRB2、OPRM1、BCL2、BAX、CASP3、CASP8、TGFB1、PON1 |
| mol03 | sitosterol | ESR1、HSD11B2、G6PD、NR3C1、APP、PPARG |
| mol04 | Stigmasterol | ESR1、NOS3、NOS2、PPARG、PTGS2、SLC6A3、ADRB2、APP AKR1B1、LTA4H、MAOA、、G6PD ADRB1、HTR2A、CHRM2、NR3C1、HSD11B2 |
| mol05 | cholesterol | ESR1 |
| mol06 | acacetin | NOS2、ESR1、PPARG、PTGS2、PTPN1、GSK3B、HSP90AA1、ADRB2、RELA、BCL2、CDKN1A、BAX、CASP3、TP53、CASP8、FASN、FASLG |
| mol07 | Isaindigodione | NOS2、ESR1、PTGS2、GSK3B、HSP90AA1 |
| mol08 | 2-O-beta-D-glucopyranosyl-2H-1,4-benzoxazin-3(4H)-one | NOS2、ESR1、PPARG、PTGS2、GSK3B、HSP90AA1 |
| mol09 | Eupatorin | NOS2、ESR1、PPARG、PTGS2、PTPN1、GSK3B、HSP90AA1 |
| mol10 | 3-[[(2R,3R,5R,6S)-3,5-dihydroxy-6-(1H-indol-3-yloxy)-4-oxooxan-2-yl]methoxy]-3-oxopropanoic acid | NOS2、ESR1、PTGS2、PTPN1、GSK3B、HSP90AA1 |
| mol11 | Dinatin | NOS2、ESR1、PPARG、PTGS2、PTPN1、GSK3B、HSP90AA1、NOS3 |
| mol12 | (-)-taxifolin | NOS2、ESR1、PPARG、PTGS2、PTPN1、GSK3B、HSP90AA1 |
| mol13 | bis[(2R)-2-ethylhexyl] benzene-1,2-dicarboxylate | ADRB2 |
| mol14 | 24-Ethylcholest-4-en-3-one | ESR1 |
| mol15 | quindoline | ESR1、PTGS2 |
| mol16 | hydroxyindirubin | NOS2、ESR1、PPARG、PTGS2、PTPN1、GSK3B、HSP90AA1 |
| mol17 | poriferast-5-en-3beta-ol | ESR1 |
| mol18 | Ineketone | ESR1 |
| mol19 | Sinoacutine | NOS2、ESR1、PTGS2、NOS3、OPRM1、GSK3B、HSP90AA1 |
| mol20 | Indigo | PTGS2 |
| mol21 | (2Z)-2-(2-oxoindolin-3-ylidene)indolin-3-one | NOS2、ESR1、PTGS2、GSK3B、HSP90AA1 |
| mol22 | 2-(9-((3-methyl-2-oxopent-3-en-1-yl)oxy)-2-oxo-1,2,8,9-tetrahydrofuro[2,3-h]quinolin-8-yl)propan-2-yl acetate | NOS2、ESR1、PTGS2、GSK3B、HSP90AA1、KCNMA1 |
| mol23 | adenosine | NOS2、ESR1、PTGS2、NOS3、PTPN1、ADORA2A |
| mol24 | Liquiritigenin | NOS2、ESR1、PPARG、PTGS2、ADRB2、GSK3B、HSP90AA1、PTPN1 |
| mol25 | (E)-2-[(3-indole)cyanomethylene-]-3-indolinone | NOS2、ESR1、PTGS2、GSK3B、HSP90AA1 |
| mol26 | neohesperidin_qt | NOS2、ESR1、PPARG、PTGS2、PTPN1、GSK3B、HSP90AA1 |
| mol27 | rosasterol | ESR1 |
| mol28 | Sinensetin | NOS2、ESR1、PPARG、PTGS2、PTPN1、ADRB2、HSP90AA1 |
| mol29 | Stigmasta-5,22-diene-3beta,7alpha-diol | ESR1 |
| mol30 | Stigmasta-5,22-diene-3beta,7beta-diol | ESR1 |
| mol31 | 6-(3-oxoindolin-2-ylidene)indolo[2,1-b]quinazolin-12-one | ESR1、PTGS2 |
| mol32 | (E)-3-(3,5-dimethoxy-4-hydroxy-benzylidene)-2-indolinone | NOS2、ESR1、PPARG、PTGS2、PTPN1、GSK3B、HSP90AA1 |
| mol33 | (E)-3-(3,5-dimethoxy-4-hydroxyb-enzylidene)-2-indolinone | NOS2、ESR1、PPARG、PTGS2、PTPN1、ADRB2、GSK3B、HSP90AA1 |
| mol34 | Indican, plant | NOS2、ESR1、PPARG、PTGS2、HSP90AA1 |
| mol35 | Isaindigotone | NOS2、ESR1、PPARG、PTGS2、PTPN1、GSK3B、HSP90AA1 |
| mol36 | Glucobrassicin-1-Sulfonate_qt | ESR1、PTGS2 |
| mol37 | isovitexin | PTGS2、PTPN1、RELA、TNF、NOS2 |
| mol38 | Bifendate | ESR1、PTGS2、GSK3B、HSP90AA1、KCNMA1、NOS2 |
| mol39 | Friedelin | ESR1 |
| mol40 | fernenol | ESR1 |
| mol41 | Luteolinidin | NOS2、ESR1、PPARG、PTGS2、PTPN1、HSP90AA1 |
| mol42 | simiarenol | ESR1 |
| mol43 | isoarborinol | ESR1 |
| mol44 | isochlorogenic,acid | PTPN1 |
| mol45 | Coixol | NOS2、PTGS2、NOS3 |
| mol46 | Mandenol | PPARG、PTGS2、NOS3 |
| mol47 | Ethyl oleate (NF) | PPARG、NOS3 |
| mol48 | 1-methoxyacetylshikonin | NOS2、ESR1、PPARG、PTGS2、NOS3、PTPN1、GSK3B、HSP90AA1、KCNMA1 |
| mol49 | Propionylshikonin | NOS2、ESR1、PPARG、PTGS2、GSK3B、HSP90AA1 |
| mol50 | acetylshikonin | NOS2、ESR1、PPARG、PTGS2、GSK3B、HSP90AA1、NOS3、PTPN1 |
| mol51 | Arnebin 7 | NOS2、ESR1、PPARG、PTGS2、NOS3、ADRB2、GSK3B |
| mol52 | Isoarnebin 4 | NOS2、ESR1、PPARG、PTGS2、PTPN1、GSK3B |
| mol53 | lithospermidin A | NOS2、ESR1、PTGS2、HSP90AA1 |
| mol54 | shikonofuran C | NOS2、ESR1、PPARG、PTGS2、GSK3B、HSP90AA1 |
| mol55 | shikonofuran B | NOS2、ESR1、PPARG、PTGS2、GSK3B、HSP90AA1 |
| mol56 | arnebinol | ESR1、PTGS2、SLC6A3、ADRB2、HSP90AA1 |
| mol57 | arnebinone | NOS2、ESR1、PTGS2、NOS3 |
| mol58 | 5-[(E)-5-(3-furyl)-2-methyl-pent-2-enyl]-2,3-dimethoxy-p-benzoquinone | NOS2、ESR1、PPARG、PTGS2、NOS3 |
| mol59 | Lithospermidin B | NOS2、ESR1、PTGS2 |
| mol60 | quercetin | NOS2、ESR1、PPARG、PTGS2、PTPN1、GSK3B、HSP90AA1、AKR1B1、ADRB2、MMP3、NOS3、RELA、EGFR、AKT1、VEGFA、BCL2、BCL2L1、CDKN1A、BAX、MMP2、MMP9、MAPK1、IL10、EGF、TNF、IL6、CASP3、TP53、NFKBIA、ODC1、CASP8、SOD1、MMP1、HIF1A、STAT1、HSPA5、HMOX1、CYP1A2、ICAM1、IL1B、CCL2、SELE、VCAM1、CXCL8、PRKCB、HSPB1、TGFB1、IL2、NR1I2、PLAT、SERPINE1、IFNG、ALOX5、PTEN、IL1A、MPO、NCF1、ABCG2、GSTP1、NQO1、PARP1、AHR、PSMD3、SLC2A4、CXCL2、NR1I3、INSR、PPARA、PPARD、CRP、CXCL10、SPP1、RUNX2、CTSD、IGFBP3、IGF2、PON1、HK2、GSTM1 |
| mol61 | isorhamnetin | NOS2、ESR1、PPARG、PTGS2、PTPN1、GSK3B、HSP90AA1、PPARD、AKR1B1、NOS3、RELA、NCF1 |
| mol62 | kaempferol | NOS2、ESR1、PPARG、PTGS2、PTPN1、GSK3B、HSP90AA1、NOS3、CHRM2、RELA、AKT1、BCL2、BAX、TNF、CASP3、MAPK8、MMP1、STAT1、HMOX1、CYP1A2、ICAM1、SELE、VCAM1、NR1I2、ALOX5、GSTP1、AHR、PSMD3、SLC2A4、NR1I3、INSR、GSTM1、AKR1C3 |
| mol63 | soyasponin I | NOS2、ESR1、PPARG、PTGS2、PTPN1、GSK3B、HSP90AA1 |
| mol64 | N-[6-(9-acridinylamino)hexyl]benzamide | NOS2、NOS3 |
| mol65 | kaikasaponinIII_qt | ESR1 |
| mol66 | quercetin-3'-methyl ether | ESR1 |
| mol67 | quercetin | ESR1 |
| mol68 | Mairin | NOS2、ESR1、PPARG、PTGS2、PTPN1、GSK3B、HSP90AA1、CAT |
| mol69 | sitosterol | ADRB1、PTGS2、SLC6A3、ADRB2、MAOA、CHRM2、RELA、AKT1、BCL2、BAX、MAPK1、TNF、NFKBIA、ICAM1、IL2、PTEN |
| mol70 | kaempferol | NOS2、ESR1、PTGS2、GSK3B、HSP90AA1 |
| mol71 | (+)-catechin | ESR1、PPARG、GSK3B、HSP90AA1 |
| mol72 | paeonol | ESR1、PTGS2 |
| mol73 | Paeonolide | ESR1 |
| mol74 | paeonoside | HSP90AA1 |
| mol75 | trametenolic acid | ESR1 |
| mol76 | Cerevisterol | ESR1 |
| mol77 | ergosta-7,22E-dien-3beta-ol | ESR1 |
| mol78 | Ergosterol peroxide | ESR1 |
| mol79 | hederagenin | ESR1、CHRM2、ADH1B、PTGS2、PPARG、NOS3 |
| mol80 | wogonin | NOS2、ESR1、PPARG、PTGS2、PTPN1、GSK3B、HSP90AA1、ADRB2、RELA、AKT1、BCL2、CDKN1A、BAX、TNF、IL6、CASP3、TP53、MMP1、CCL2、FN1、CXCL8 |
| mol81 | Ammidin | NOS2、ESR1、PPARG、PTGS2、GSK3B |
| mol82 | isoimperatorin | NOS2、ESR1、PTGS2 |
| mol83 | Marmesin | NOS2、ESR1、PTGS2、ADRB2、CHRM2、PTPN1、LTA4H、HSP90AA1 |
| mol84 | Phellopterin | NOS2、ESR1、PPARG、PTGS2、NOS3、PTPN1、ADRB2、GSK3B、HSP90AA1 |
| mol85 | Prangenidin | NOS2、ESR1、PTGS2、GSK3B、HSP90AA1 |
| mol86 | methyl icosa-11,14-dienoate | ESR1、PPARG、NOS3 |
| mol87 | heptadeca-1,8-dien-4,6-diyn-3,10-diol | PPARG、PTGS2 |
| mol88 | 11-hydroxy-sec-o-beta-d-glucosylhamaudol_qt | NOS2、ESR1、PTGS2、PTPN1、GSK3B |
| mol89 | 3'-O-Acetylhamaudol | NOS2、ESR1、PPARG、PTGS2、PTPN1、GSK3B |
| mol90 | anomalin | NOS2、ESR1、PTGS2 |
| mol91 | divaricatacid | NOS2、ESR1、PTGS2、GSK3B |
| mol92 | divaricatol | NOS2、ESR1、PPARG、PTGS2、PTPN1、GSK3B |
| mol93 | ledebouriellol | NOS2、ESR1、PPARG、PTGS2 |
| mol94 | phelloptorin | NOS2、ESR1、PPARG、PTGS2、PTPN1、GSK3B |
| mol95 | 5-O-Methylvisamminol | NOS2、ESR1、PTGS2、NOS3、ADRB2、OPRM1、GSK3B |
| mol96 | Decursin | NOS2、ESR1、PPARG、PTGS2、PTPN1、ADRB2GSK3B、HSP90AA1 |
| mol97 | lignoceric acid | PPARG、NCF1 |
| mol98 | catapol_qt | NOS3 |
| mol99 | Rehmaionoside C | ESR1 |
| mol100 | catalpol | BCL2、NOS2、CASP3、SOD1 |
| mol101 | Epi-Friedelanol | ESR1 |
| mol102 | 3,4,5-trihydroxybenzoic acid | PTGS2、ESR1、NOS2、PPARG、PTPN1、GSK3B、HSP90AA1、CASP3、TP53、FASN、FASLG |
| mol103 | ellagic acid | ESR1、HSP90AA1、RELA、VEGFA、CDKN1A、MMP2、MMP9、、IGF2、NFKBIA、CXCL8、PRKCB、GSTP1、GSTM1 |
| mol104 | baicalein | NOS2、ESR1、PPARG、PTGS2、PTPN1、GSK3B、HSP90AA1、RELA、AKT1、VEGFA、BCL2、BAX、MMP9、CASP3、TP53、HIF1A、MPO、AHR、IGF2、CYCS、ALOX12 |
| mol105 | Baicalin | PTPN1、GSK3B |
| mol106 | Spinasterol | ESR1 |
| mol107 | campest-5-en-3beta-ol | ESR1 |
| mol108 | (2R,3R)-4-methoxyl-distylin | NOS2、ESR1、PPARG、PTGS2、PTPN1、GSK3B、HSP90AA1 |
| mol109 | stigmast-7-en-3-ol | ESR1 |
| mol110 | paeonin,a | NOS2、ESR1 |
| mol111 | paeonin,a_qt | NOS2、PTGS2、NOS3 |
| mol112 | paeonin,b | ESR1 |
| mol113 | paeonin,b_qt | NOS2、、PTGS2、NOS3 |
| mol114 | paeoniflorin | TNF、IL6、LBP |
| mol115 | luteolin | NOS2、ESR1、PPARG、PTGS2、PTPN1、HSP90AA1、GSK3B、RELA、EGFR、AKT1、VEGFA、BCL2L1、CDKN1A、MMP2、MMP9、MAPK1、IL10、TNF、IL6、CASP3、TP53、NFKBIA、MDM2、APP、MMP1、PCNA、HMOX1、ICAM1、IL2、IFNG、IL4、GSTP1、SLC2A4、INSR |
| mol116 | Skimmianin | NOS2、ESR1、PPARG、HSP90AA1、GSK3B |
| mol117 | 3'-O-methyl taxifolin | NOS2、ESR1、PPARG、PTGS2、PTPN1、GSK3B、HSP90AA1 |
| mol118 | Dasycarpamin | NOS2、ESR1、PPARG、PTGS2、NOS3、CHRM2、SLC6A3、ADRB2、GSK3B、PTPN1、OPRM1、HSP90AA1 |
| mol119 | O-ethylnor-γ-fagarine | ESR1、HSP90AA1 |
| mol120 | Dictamdiol A | NOS2、ESR1、PTGS2、NOS3 |
| mol121 | preskimmianine | NOS2、ESR1、PPARG、PTGS2、NOS3、CHRM2、SLC6A3、ADRB2、OPRM1、GSK3B、HSP90AA1 |
| mol122 | tirucallane | ESR1 |
| mol123 | 9alpha-hydroxyfraxinellone-9-o-beta-d-glucoside | ESR1 |
| mol124 | psoralen | ESR1 |
| mol125 | ursolic acid | RELA、STAT3、VEGFA、BCL2、BCL2L1、CDKN1A、BAX、MMP2、MMP9、TNF、IL6、NOS2、CDK6、CASP3、TP53、MAPK8、PTGS2、NFKBIA、CASP8、FASN、MMP1、MMP3、FGF2、MMP10、ICAM1、IL1B、SELE、CSF2、NOS3、PTPN1、INPPL1、CCND2、FASLG、CASP1 |
| mol126 | p-coumaric acid | NOS2、PTGS2、NOS3、MAOA |
| mol127 | 2-hydroxy-3-methylanthraquinone | NOS2、ESR1、PPARG、PTGS2、NOS3、HRH1、HTR2A、HTR2C、CHRM2、PTPN1、SLC6A3、ADRB2、OPRM1、GSK3B、HSP90AA1、LTA4H、CASP3 |
| mol128 | E-6-O-p-methoxycinnamoyl scandoside methyl ester_qt | NOS2、ESR1、PPARG、PTGS2、PTPN1、GSK3B、HSP90AA1 |
| mol129 | Oleanolic acid-28-O-beta-D-glucopyranoside | ESR1 |
| mol130 | scandoside_qt | NOS2、PTGS2、NOS3 |
| mol131 | Poriferasterol | ESR1 |
| mol132 | scandoside_qt | NOS2、PPARG、PTGS2、NOS3 |
| mol133 | 3-Epioleanolic acid | ESR1 |
| mol134 | 2-methoxy-3-methyl-9,10-anthraquinone | NOS2、ESR1、PPARG、PTGS2、NOS3、HRH1、HTR2A、HTR2C、CHRM2、PTPN1、SLC6A3、ADRB2、OPRM1、GSK3B、HSP90AA1 |
| mol135 | rutin | RELA、TNF、IL6、NOS2、CASP3、SOD1、CAT、IL1B、CXCL8、PRKCB、ALOX5、GSTP1、INS、FCER2、ITGB2 |
| mol136 | POlyPhyllin 1 | HSD11B2、CALCRL、NR3C1、G6PD、NTSR1、FKBP5、GLB1 |
| mol137 | Daucosterol | GLB1、HSD11B2、G6PD、NR3C1、PPARG、APP、FKBP5 |
| mol138 | Daucosterol | HSD11B2、NR3C1、G6PD、PPARG |
| mol139 | Creatinine | AOC3 |
| mol140 | POlyPhyllin 3 | HSD11B2、FKBP5、NR3C1、G6PD、CALCRL、NTSR1、APP、CCL5 |
| mol141 | α- Ecdysterone | HSD11B2、G6PD、GLB1、NR3C1 |
| mol142 | β- Ecdysterone | HSD11B2、G6PD、GLB1、NR3C1 |
| mol143 | methylprotodioscin | HSD11B2、CCL5、NTSR1、CALCRL、NR3C1、G6PD |
| mol144 | Methylprotodioscin_qt | HSD11B2、CALCRL、NR3C1、G6PD、CCL5、NTSR1、FKBP5 |
