# Supplementary material for: Systems Pharmacology Approach and Experiment Evaluation Reveal Multidimensional Treatment Strategy of LiangXueJieDu Formula for Psoriasis
Source: Front Pharmacol. 2021 Jun 8;12:626267. doi: 10.3389/fphar.2021.626267 (PMC8217833; doi:10.3389/fphar.2021.626267)
Supplement: Supplementary file 4 [file Table4.DOCX]

**Supp Table S4 The crucial target protein associated pathways**

| **Name** | **Attribute** | **Degree** | **BetweennessCentrality** |
| --- | --- | --- | --- |
| PI3K-Akt signaling pathway | pathway | 27 | 0.25701163 |
| TNF signaling pathway | pathway | 21 | 0.20062182 |
| MAPK signaling pathway | pathway | 16 | 0.09578236 |
| Toll-like receptor signaling pathway | pathway | 15 | 0.06058911 |
| NOD-like receptor signaling pathway | pathway | 13 | 0.04278691 |
| Salmonella infection | pathway | 13 | 0.06795168 |
| Ras signaling pathway | pathway | 13 | 0.04548686 |
| Apoptosis | pathway | 12 | 0.06625926 |
| Calcium signaling pathway | pathway | 12 | 0.10677827 |
| NF-kappa B signaling pathway | pathway | 11 | 0.05329387 |
| T cell receptor signaling pathway | pathway | 11 | 0.04170324 |
| Jak-STAT signaling pathway | pathway | 11 | 0.05354109 |
| cAMP signaling pathway | pathway | 10 | 0.08434361 |
| ErbB signaling pathway | pathway | 8 | 0.0194067 |
| Cell cycle | pathway | 8 | 0.03003015 |
| VEGF signaling pathway | pathway | 7 | 0.04944741 |
| Fc epsilon RI signaling pathway | pathway | 7 | 0.01242478 |
| B cell receptor signaling pathway | pathway | 6 | 0.02634752 |
| Wnt signaling pathway | pathway | 6 | 0.02948088 |
| Arachidonic acid metabolism | pathway | 5 | 0.08552317 |
